# Query Large Scale Microarray Compendium Datasets Using a Model-Based Bayesian Approach with Variable Selection

**DOI:** 10.1371/journal.pone.0004495

**Published:** 2009-02-13

**Authors:** Ming Hu, Zhaohui S. Qin

**Affiliations:** Center for Statistical Genetics, Department of Biostatistics, School of Public Health, University of Michigan, Ann Arbor, Michigan, United States of America; University of Liverpool, United Kingdom

## Abstract

In microarray gene expression data analysis, it is often of interest to identify genes that share similar expression profiles with a particular gene such as a key regulatory protein. Multiple studies have been conducted using various correlation measures to identify co-expressed genes. While working well for small datasets, the heterogeneity introduced from increased sample size inevitably reduces the sensitivity and specificity of these approaches. This is because most co-expression relationships do not extend to all experimental conditions. With the rapid increase in the size of microarray datasets, identifying functionally related genes from large and diverse microarray gene expression datasets is a key challenge. We develop a model-based gene expression query algorithm built under the Bayesian model selection framework. It is capable of detecting co-expression profiles under a *subset* of samples/experimental conditions. In addition, it allows linearly transformed expression patterns to be recognized and is robust against sporadic outliers in the data. Both features are critically important for increasing the power of identifying co-expressed genes in large scale gene expression datasets. Our simulation studies suggest that this method outperforms existing correlation coefficients or mutual information-based query tools. When we apply this new method to the *Escherichia coli* microarray compendium data, it identifies a majority of known regulons as well as novel potential target genes of numerous key transcription factors.

## Introduction

Genome-wide expression analysis with DNA microarray technology [1], [2]. has become an indispensable tool in genomics research [3]. Increased accessibility, lowered cost and improved technology result in more comprehensive studies, under more diverse conditions and a rapid expansion of available gene expression data. This presents an important resource for mining biological information. A particular example is the so-called microarray compendium in which gene expression profiles were surveyed in hundreds of samples which were treated under diverse biological conditions [4]–[6]. Data generated from such studies is highly informative. However, due to heterogeneity, finding biological insight from such datasets proves a major challenge. Scalable and effective mining tools capable of extracting knowledge from diverse and noisy information sources are critically needed [7].

An effective data mining tool for gene expression microarray data is to infer relatedness among genes based on their expression profile, a tactic referred to as the “guilt by association” (GBA) principle [8]–[12]. The underlying hypothesis is that functionally related genes, such as transcription factor (TF) and its regulated genes—regulon —tend to display correlated gene expression patterns. For example, Mootha et al. [13] proposed the “neighborhood analysis algorithm” to identify “neighboring” genes that share correlated expression profiles with genes of interest. Various measurements such as Pearson correlation, Spearman's rank correlation, Kendall's 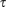 and mutual information [14] have been used to assess the strength of the correlation. Recently, much interest has been generated on genome-wide regulatory network inference [15], where pairwise regulatory relationships among genes need to be predicted. As an example, Faith et al. [4] developed the context likelihood of relatedness (CLR) algorithm to identify regulatory interactions.

Although successful in analyzing small datasets, the above mentioned correlation or distance measures will be less helpful for searching large datasets, such as microarray compendium data. This is because for most functionally related genes, tight correlation only occurs under specific experimental conditions. Therefore global correlation measures taken across diverse experimental conditions will be significantly reduced, and thus undermine its ability to recognize functional related genes. Given the microarray compendium scenario, we hypothesized that statistically significant correlation can still be detected using microarray, but strong correlation will be confined to a subset of samples/experimental conditions. Under this hypothesis, it is highly desirable to develop a query tool that can automatically recognize a subset of conditions under which the query gene and its targets share tightly correlated expression profiles.

**Figure 1:**
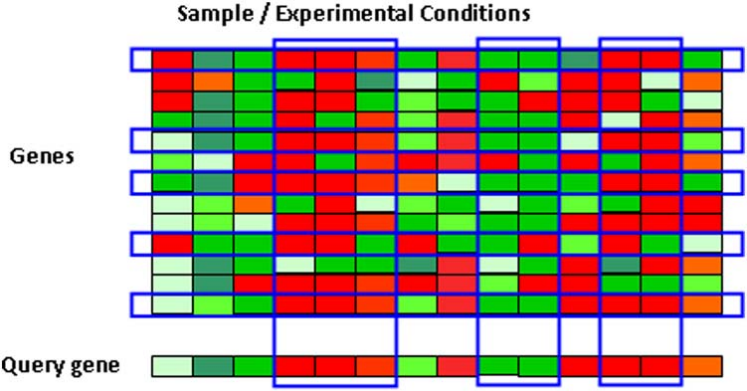
Illustration of the model-based gene expression query algorithm. Each row represents a gene, and each column represents a sample/experimental condition. The query gene is at the bottom. The Blue boxes indicate the collection of genes and experimental conditions in which co-expression with the query gene is observed.

In this manuscript, we design a model-based query algorithm capable of detecting significantly correlated expression patterns that are restricted to a subset of experimental conditions. See Figure 1 for an illustration of our scheme. This approach not only predicts functionally related genes, it also allows one to discover under which experimental conditions such co-expression occurs. The proposed query tool will provide researchers with a much needed device to explore the rich resources of vast microarray databases available. This model is inspired by the Bayesian Partition with Pattern Selection (BPPS) model designed to identify functionally related proteins [16]. Our proposed method is related to bi-clustering [17]–[21] since we consider both genes and samples/experimental conditions. However, bi-clustering is unsupervised, which is different from the supervised pattern matching procedure we propose. Qian et al. [22] introduced a pairwise query algorithm for gene expression data based on a Smith-Waterman type local alignment algorithm [23]. However, that algorithm is designed for querying time-course gene expression data only, and is generally not applicable to datasets where the experimental conditions are unrelated. Dhollander et al. [24] introduced a model-based query-driven module discovery tool—QDB, but it is aimed at performing informed bi-clustering instead of pattern matching, and it does not take into account the complex correlation patterns such as inverse patterns. Owen et al. [25] proposed a score-based search algorithm called gene recommender (GR) to find genes that are co-expressed with a given set of genes using data from large microarray datasets. GR first selects a subset of experiments in which the query genes are most strongly co-regulated. Hence multiple query genes are required.

## Methods

### 1. Statistics model

We propose a model-based query tool for gene expression data. The goal is to identify genes that share correlated expression profiles with a particular gene such as a key TF.

The entire microarray compendium can be represented as a matrix, where each row represents a gene and each column represents an experimental condition. We are hoping to identify a subset of rows (genes) and a subset of columns (conditions) such that these genes show co-expression with the query gene under the selected conditions. This procedure is similar to placing binary labels on all rows and columns. Finding the maximum likelihood estimator is often a good solution to such a statistical inference problem. However, the large number of rows and columns make it impossible for us to enumerate all possible combinations. We therefore employ the Markov chain Monte Carlo strategy to guide us for a more efficient search. The statistical model and computational algorithm is as follows (more details can be found in the supplementary Material S1).

Suppose there is a database containing expression levels of *N* genes across *M* different experimental conditions. Each gene is represented by an expression vector 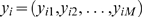 that can be summarized as a data matrix 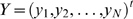. Given a particular query expression profile 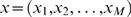, we want to identify all genes that share similar expression patterns across a subset of experimental conditions. To do this, we define a difference vector as 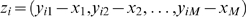, and use 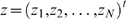 as the input data for our inference. We introduce a row indicator vector 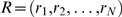 and a column indicator vector 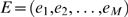, *r_i_* = 1 indicates that gene *i* in the database is functionally related to the query gene and 0 otherwise. *e_j_* = 1 indicates that co-expression occurs at the *j* ^th^ experimental condition (foreground) and 0 otherwise (background). We assume that the differences between a related gene and the query gene at the foreground columns follow normal distributions 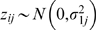. The remainder of *Z* is assumed to follow background normal distributions 
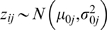
 where 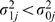. Let 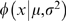 represents the probability density function of normal distribution with mean *μ* and variance 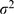. The overall likelihood can be expressed as:
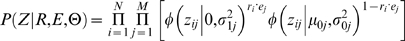
where 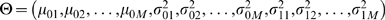. We adopt standard conjugate priors for these model parameters [26].
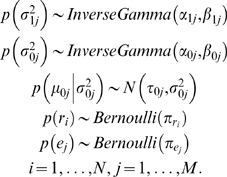



Specification of these prior distributions can be found in the Supplementary Material S1.

Parameters of interest are the two indicator vectors *R* and *E*. *Θ* is regarded as the nuisance parameter and is integrated out to simplify the computation [27]. We use the Gibbs sampler [28], [29] to sample *R* and *E* from the posterior distributions. To be specific, our algorithm will cycle through all rows and columns sequentially, flip the indicator variables of each row or column, and then decide whether to accept the change based on the Bayes factor calculated. The joint distributions can be derived as follows:
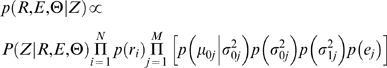



After integrating out nuisance parameters, we get full conditional distributions of *R* and *E*, which are Bernoulli distributions. The details can be found in Formula 1 in the Supplementary Formula S1 document.

The detailed procedure of our algorithm is as follows.

1. Initialization: randomly assign row and column indicators to be either one or zero. Calculate the differences 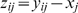.
2. Cycle through all rows and columns sequentially 50 times. At each cycle, draw the indicator for each row and column from the full conditional distributions. The result with the highest log likelihood during the 50 cycles is recorded.
3. Repeat the cycle ten times, and report the result with the highest log likelihood from all runs.

In the initialization step, the row and column indicators can be assigned randomly. In practice, one can simply assign 1 to the top half of rows and to the first half of columns and 0 to the rest of rows and columns. If there is additional information suggesting certain genes (rows) are targets (or non-targets) of the TF, it is recommended that the indicator 1 (or 0) be assigned to those genes and the same for the experimental conditions.

### 2. Add linear factor

In the previous model, we require that the target genes and the query gene share similar expression levels in selected experimental conditions. This is restrictive since functionally related genes may display the same expression pattern but differ in absolute quantity. To capture this, we extend our model to allow the expression levels of the target gene and the query gene to differ by a constant factor. That is, their expression profiles are proportional to each other: *y_ij_* = *a_i_ x_j_*. Here *a_i_* is a linear transformation factor for gene *i*. *a_i_* can be either positive or negative indicating positive or negative correlation respectively. After normalization, we estimate the linear transformation factor *a_i_* using least square without intercept. To keep our model simple and avoid over-fitting, we restrict the linear factor to be significantly different from 0 and 1. The estimation step is made at the beginning of each cycle based on the most recently updated column indicators.

### 3. Allow cell-level noise

In the aforementioned models, genes selected are mandated to have similar expression profiles up to a constant factor under a subset of experimental conditions. Hence the chosen rows and columns in the original data matrix form a solid block when combined. This may still be too restrictive because a few sporadic cells in the block may deviate from the corresponding values in the query profile. Possible reasons that may cause such discrepancy are experimental artifacts, measurement errors, or substructures in the co-expression pattern. To account for this, we introduce an additional binary indicator variable, *c_ij_*, for each cell in this block to indicate whether this particular gene/experimental condition combination should be treated as background. This additional step allows us to identify significant but imperfect patterns. Adding this additional parameter, the overall likelihood is modified as follows:
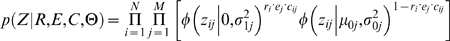



We use a Bernoulli distribution as the prior for *c_ij_*,
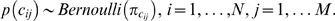



The prior for this new indicator variable will be set such that only a small fraction of cells is allowed to be treated as background.

After integrating out nuisance parameters, the full conditional distributions of all model parameters can be obtained similarly as before. The details can be found in formula 2 in the Supplementary Formula S1 document.

## Results

The aforementioned algorithm has been implemented in a C++ program named BEST (Bayesian Expression Search Tool). To test its performance, we applied it to a series of synthetic datasets as well as to the real *Escherichia coli* microarray compendium dataset [30]. In addition to BEST, we also tested well-established query tools based on Pearson, Spearman correlation coefficients, Kendall's 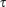, mutual information [14] and the model-based query-driven module discovery tool—QDB [24].

### 1. Synthetic datasets

All simulated data contained 100 rows (genes) and 50 columns (experimental conditions). Around 20% of the 100 genes were randomly assigned as the “target” genes. Let *T* represent the total number of target genes in a dataset. To mimic the scenarios that gene expression correlation only presents in a subset of experimental conditions, we separated the 50 columns into foreground and background and require that correlated expression profiles between the query gene and the target genes can only be observed among foreground columns. To assess the impact of the proportion of foreground columns on the effectiveness of identifying target genes, we tested four different settings: 100%, 75%, 50% and 25% of columns were selected as foreground. At each foreground column, the expression profiles of the query gene and *T* target genes were generated from a *T*+1 dimensional multivariate normal distribution with mean zero and variance-covariance matrix Σ. The correlation coefficient between the query gene and each target gene was set to be 0.95.

**Figure 2:**
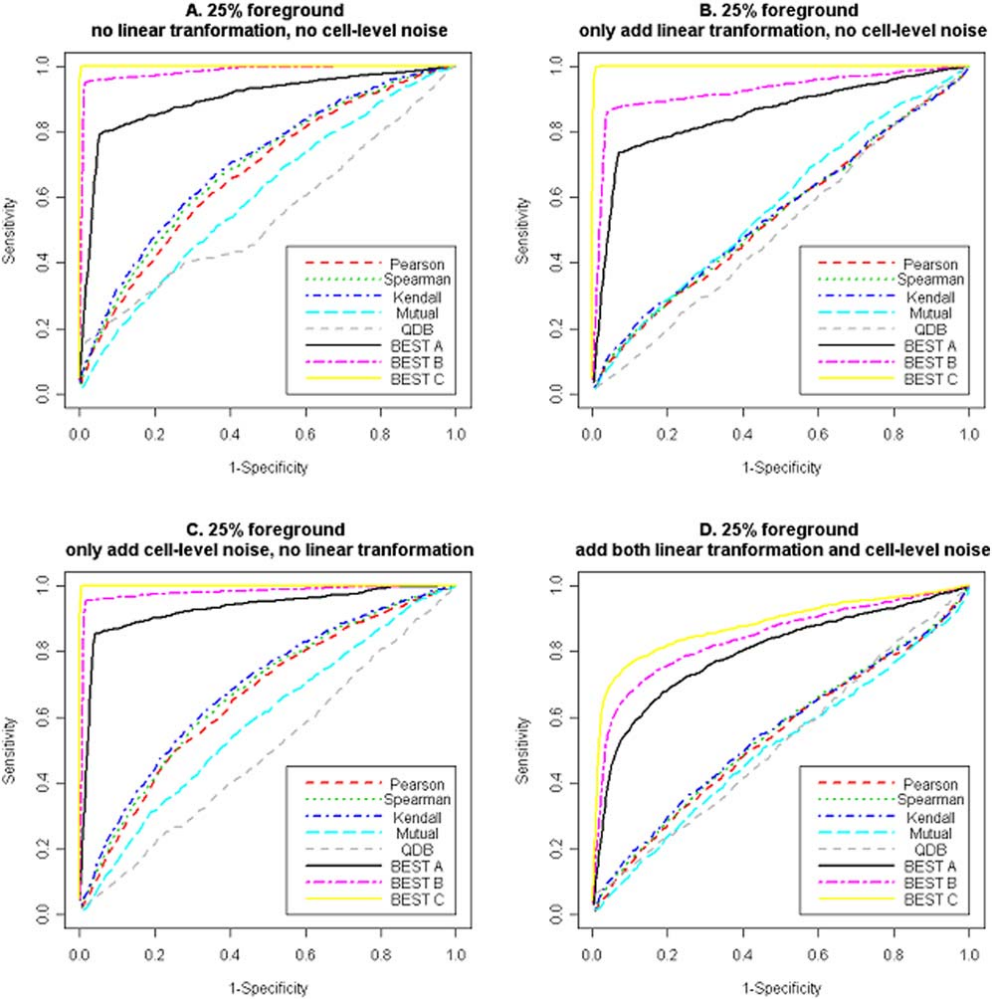
ROC curves for various query methods when applying to synthetic datasets simulated under different settings and when there are 25% foreground columns. BEST A default setting; BEST B allowing exclusion of individual cells from the foreground; BEST C fixing the indicator variables of five true target genes and five true experimental conditions as 1. A. No linear transformation nor cell-level noise. B. With linear transformation only. C. With cell-level noise only. D. With both linear transformation and cell-level noise.

The remaining expression profiles were generated independently from a uniform distribution between −4 and 4. To mimic the noisy nature of the microarray data, we included the following additional settings: randomly add linear transformations to 50% of the target genes (the linear transformation factors were randomly picked from (−2, −1, −0.5, 0.5, 2); randomly add additional noise (±5) to 10% of the expression values of target genes in foreground columns to mimic outliers caused by experimental artifacts. We also considered settings in which neither or both of these two complications were present. The combination of these four scenarios with the four different proportions of foreground columns mentioned above resulted in 16 different testing cases. We generated 50 simulated datasets for each of the 16 cases, and tested all query methods on each dataset to identify target genes. To compare performance, we sorted the 100 genes using the relatedness measures adopted in each method and found the proportions of true positives among the top *T* genes. The means and standard deviations of these proportions were summarized in Table 1. We also produced Receiver Operating Characteristic (ROC) curves for all methods under all simulation settings. ROC curves obtained from the most challenging scenario, where only 25% of the columns are foreground, are shown in Figure 2. ROC curves obtained from other simulation settings can be found in Supplementary Figures S1, S2, and S3. The areas under the curve (AUC) of these ROC curves were summarized in Supplementary Table S1.

**Table 1:** Performance^1^ comparison among various methods for querying simulated microarray gene expression dataset. Best results are displayed in bold.

Case	Sub-case*	Pearson^a^	Spearman^b^	Kendall^c^	QDB^d^	Mutual^e^	BEST A^f^	BEST B^g^	BEST C^h^
Case 1:	I	**1 (0)**	**1 (0)**	**1 (0)**	**1 (0)**	**1 (0)**	**1 (0)**	**1 (0)**	**1 (0)**
100%	II	0.67 (0.12)	0.68 (0.12)	0.68 (0.12)	0.59 (0.13)	**1 (0.01)**	**1 (0)**	**1 (0)**	**1 (0)**
foreground	III	**1 (0)**	**1 (0)**	**1 (0)**	**1 (0)**	**1 (0)**	**1 (0)**	**1 (0)**	**1 (0)**
	IV	0.62 (0.09)	0.70 (0.09)	0.70 (0.09)	0.51 (0.11)	0.78 (0.08)	0.97 (0.04)	**0.98 (0.03)**	**0.98 (0.03)**
Case 2:	I	0.89 (0.10)	0.96 (0.05)	0.99 (0.03)	**1 (0)**	0.87 (0.09)	**1 (0)**	**1 (0)**	**1 (0)**
75%	II	0.66 (0.12)	0.71 (0.10)	0.70 (0.09)	0.70 (0.10)	0.81 (0.09)	**1 (0)**	**1 (0)**	**1 (0)**
foreground	III	0.91 (0.09)	0.97 (0.04)	0.99 (0.03)	**1 (0)**	0.87 (0.09)	**1 (0)**	**1 (0)**	**1 (0)**
	IV	0.61 (0.11)	0.68 (0.11)	0.70 (0.11)	0.53 (0.12)	0.70 (0.11)	**0.97 (0.04)**	**0.97 (0.04)**	**0.97 (0.04)**
Case 3:	I	0.66 (0.17)	0.73 (0.14)	0.80 (0.13)	0.97 (0.16)	0.61 (0.14)	**1 (0)**	**1 (0)**	**1 (0)**
50%	II	0.51 (0.11)	0.59 (0.11)	0.62 (0.12)	0.71 (0.13)	0.52 (0.13)	**1 (0)**	**1 (0)**	**1 (0)**
foreground	III	0.63 (0.14)	0.70 (0.13)	0.77 (0.12)	0.91 (0.25)	0.59 (0.15)	**1 (0)**	**1 (0)**	**1 (0)**
	IV	0.42 (0.12)	0.49 (0.12)	0.53 (0.11)	0.53 (0.17)	0.43 (0.16)	0.92 (0.06)	0.92 (0.06)	**0.93 (0.05)**
Case 4:	I	0.36 (0.13)	0.38 (0.12)	0.40 (0.12)	0.29 (0.29)	0.29 (0.13)	0.79 (0.34)	0.95 (0.15)	**1 (0)**
25%	II	0.25 (0.10)	0.26 (0.09)	0.28 (0.09)	0.19 (0.08)	0.27 (0.09)	0.73 (0.36)	0.86 (0.28)	**0.99 (0.02)**
foreground	III	0.34 (0.09)	0.36 (0.09)	0.38 (0.09)	0.21 (0.14)	0.29 (0.10)	0.85 (0.29)	0.95 (0.17)	**1 (0)**
	IV	0.25 (0.08)	0.26 (0.07)	0.26 (0.07)	0.22 (0.13)	0.22 (0.11)	0.57 (0.28)	0.66 (0.25)	**0.73 (0.22)**

1 Performance was measured by the proportions of true positives among the top *T* genes. *T* is the number of true positives in each simulated dataset. The mean and standard deviation of these proportions in the 50 simulated datasets were reported.

* There are four sub-cases in each of the simulated cases with the same amount of foreground columns.

Sub case I: no linear transformation, no cell-level noise;

Sub case II: only add linear transformation;

Sub case III: only add cell-level noise;

Sub case IV: add both linear transformation and cell-level noise.

a Query method using Pearson correlation coefficient.

b Query method using Spearman correlation coefficient.

c Query method using Kendall’s *τ*.

d Query method using QDB.

e Query method using mutual information.

f Query method using BEST.

g Query method using BEST allowing exclusion of individual cells from the foreground.

h Query method using BEST when fixing the indicator variables of five true target genes and five true experimental conditions as 1.

From the simulation results, we see that all methods performed perfectly when all columns were foreground and no complicated correlation was present. In subsequent cases, the performances of all methods deteriorated with the inclusion of background columns, linear transformation and additional cell level noise. We observed that BEST is robust against added noise and complications and performed the best overall. Even in the most challenging case, in which the co-expression only occurred in 25% of the 50 columns, and half of the co-expressed genes were linearly transformed plus 10% additional cell-level noise, BEST still found 57% true co-expressed genes, and the AUC was 0.79. The simulation results also indicated that the version of BEST that allows cell-level noise has 5.4% to 8.2% higher AUCs compared to the version that does not consider cell-level noise. To evaluate the impact of incorporating existing knowledge into the model, we tested another version of BEST in which we fixed the indicator variables of five real target genes and five true foreground experimental conditions as 1. We found that in the most challenging case, AUCs further increased 1.0% to 7.6% compared to the version that considers cell-level noise. The superior performance of BEST in these synthetic datasets suggested that our algorithm worked well in the context of highly heterogeneous microarray data and was robust against moderately distorted data and sporadic outliers. Our model naturally accommodates existing biological knowledge which often results in further improvement in prediction accuracy. Among others, sophisticated methods such as QDB and the method based on mutual information performed better than the rest as expected. We acknowledge that our simulation scheme do not fit QDB well since it is a model-based bi-clustering algorithm not designed for the purpose of “querying *per se*”.

### 2. Escherichia coli dataset

This dataset originally came from the study reported in [4]. The authors conducted a comprehensive survey of gene expression profiles of all *E. coli* genes using 612 Affymetrix GeneChip arrays treated with 305 different experimental conditions. The goal of that study was to construct regulatory networks and determine the relative merits of different network inference algorithms on experimental data. RMA normalized data [30] was used in this study. This dataset consisted of 4,217 genes and 305 samples. We started with TF Leucine-responsive Regulatory Protein (Lrp) as the query gene. Faith et al. [4] listed Lrp as one of three TFs that show substantial connectivity in the network mapped by CLR and recommended by the authors as an ideal test case. The *E. coli* Lrp is the best-studied member of the Lrp family, a global regulator in *E. coli* affecting the expression of many genes and operons [31]. According to RegulonDB [32], Lrp has 61 experimentally verified transcription targets. We refer to the collection of these genes as the RegulonDB target set. Faith et al. [4] predicted potential transcription targets of Lrp using CLR, a mutual information-based algorithm. There were 43 genes predicted as Lrp targets at 60% precision and one gene was predicted as a Lrp target at 80% precision.

### 2.1 Query result from 100-gene test set

**Figure 3:**
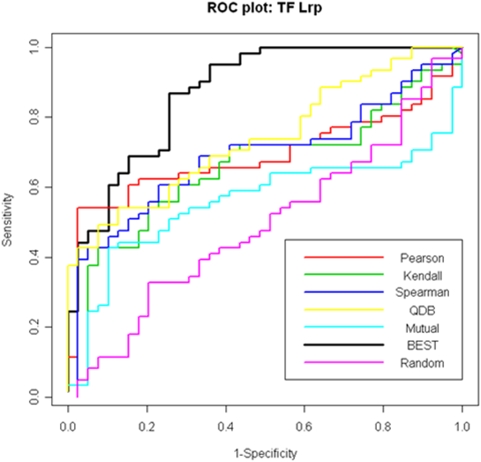
ROC curves for various query methods applying to the 100-gene test set selected from the *E. coli* microarray compendium. The area under the curves (AUC) are: Pearson correlation: 0.69; Spearman correlation: 0.69; Kendall's τ: 0.66; QDB: 0.70; Mutual information: 0.56; BEST: 0.87; Random control: 0.52.

We tested BEST on this dataset to see if it could identify known target genes of Lrp. The 61 genes in the RegulonDB target set were included as positive genes. We also included 39 *E. coli* genes which displayed the most variation across the 305 experiments and not in the RegulonDB target set as negative genes. We used the 100-gene test set to compare performance of our algorithm with other query methods based on Pearson, Spearman and Kendall correlation coefficients, mutual information and QDB. Using the default setting, BEST identified 28 target genes; 27 of them (96%) were in the RegulonDB target set (highly significant for enrichment with p-value of 1.27×10^−6^). BEST also identified 143 experimental conditions (47%) as foreground. The log-likelihood trace plot suggested rapid convergence (Supplementary Figure S4). To compare the performance of our method with others, we plotted ROC curves (Figure 3). BEST achieved an AUC of 0.87, which was significantly higher than others (≤0.70). We also randomly selected 28 genes as targets for comparison, which showed an AUC of 0.52.

**Figure 4:**
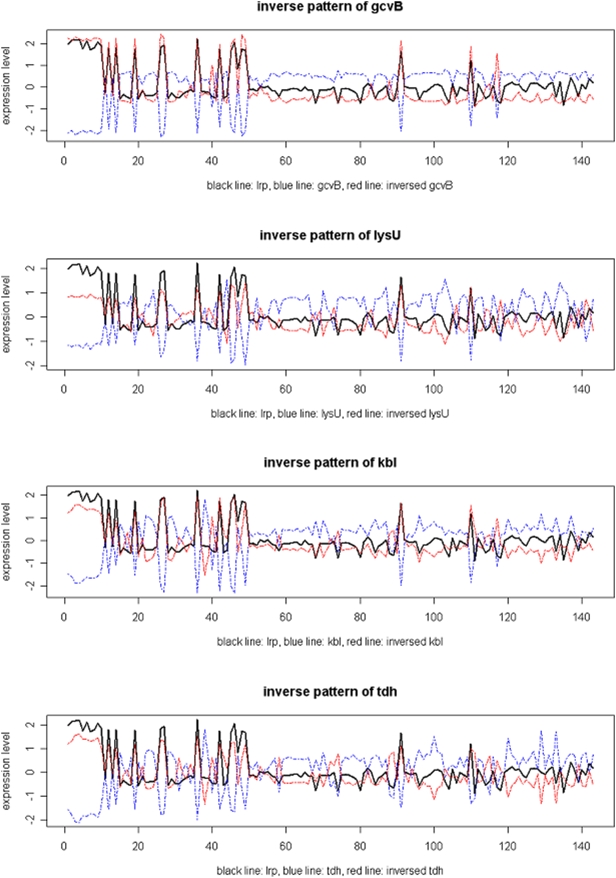
The original (blue line) and inverted (red line) expression profiles of gcvB, lysU, kbl and tdh compared to query gene Lrp. Black lines indicate the query gene—Lrp. Only the 143 foreground experimental conditions identified by BEST were shown in these plots. Results are from the 100-gene test set selected from the *E. coli* microarray compendium.

Among the 28 genes BEST identified (Supplementary Table S2), only one gene, gcvB, was not in the RegulonDB target set. gcvB is a regulatory RNA. It represses oppA, dppA, gltI and livJ expression and is regulated by gcvA and gcvR [33]. Until now there has been no evidence to suggest gcvB is regulated by Lrp. However, the trace plot (Figure 4) showed that its expression profile, after inversion, is very close to the expression profile of Lrp. Its expression profile is also very close to that of three genes found in the RegulonDB target set, lysU, kbl and tdh (Table 2 and Figure 4). Furthermore, the scan of Lrp motif pattern (Figure S5) indicates that there is a putative Lrp motif located in the intergenic region upstream of gcvB. Therefore we hypothesize that gcvB is also a target gene of Lrp (repressed by it).

**Table 2:** Information of the four genes showing inverse correlation patterns with Lrp identified by BEST when applied to the 100-gene test set selected from the *E. coli* microarray compendium.

Rank	Gene Name^a^	Log Bayes Ratio	positive/negative^b^	RegulonDB^c^	CLR^d^	motif distance^e^	Empirical p-value^f^
16	gcvB	107.8	negative			414	0.0047
23	lysU	84.52	negative	X		138	0.0044
24	kbl	81.47	negative	X		33	0.0019
25	tdh	80.09	negative	X			

All but the first one, gcvB, are in the RegulonDB target set.

a Genes displayed here are sorted by the Log Bayes ratio (target gene versus non-target gene).

b Blank indicates that the target gene shows the same pattern as the query gene. Negative indicates that the target gene shows the inversed pattern as the query gene.

c “X” indicates that the predicted gene is in the RegulonDB target set.

d “X” indicates that the gene is predicted by CLR as a target gene.

e Motif distance is defined as the distance between the start position of the gene and the closest motif in the intergenic region upstream.

f Empirical p-value indicates the significance of conservation in the current motif, which is calculated as proportion of all possible motif locations in the complete *E. coli* genome that have likelihood ratios comparing between Lrp motif and background higher than that of the current motif.

Results from BEST also suggested that Lrp is likely to actively carry out most of its regulatory role under about half of all the 305 experimental conditions tested. To verify this hypothesis, we separately calculated Pearson correlation coefficients between the expression profiles of Lrp and genes in the RegulonDB target set in the 143 foreground conditions as well as the 162 background conditions. We found that the Pearson correlation coefficient in the foreground subset was indeed significantly higher than that of the background subset. A paired t-test comparing the two sets of correlation coefficients returned a p-value of 0.0079. When restricted to the 28 genes BEST identified as targets, the difference in the matched correlation coefficients became even more significant (p-value of 1.948×10^−12^). Side-by-side box plots are shown in Supplementary Figure S6. The 305 experimental conditions were listed in Table S3 which was sorted by log Bayes ratio (larger values correspond to foreground). We found that many of the experimental conditions listed in the top portion are related to minimum media or stress which is consistent to what Faith et al.[4] found that including minimum media conditions will help identify Lrp targets.

The core of our model is the two-component Gaussian mixture for the expression levels obtained under the foreground experimental conditions. To verify this assumption, we plotted histograms of expression levels obtained under ten different experimental conditions, the top and bottom five when sorted by the Bayes ratio comparing whether an experimental condition is foreground or background. The histograms are shown in Supplementary Figure S7. As expected, we observed that the histograms from the top five experimental conditions show strong bi-modal shapes while those from the bottom five do not.

### 2.2 Query result from 300-gene test set

To evaluate whether increased number of genes being queried and change in the proportion of negative genes affect BEST's performance, we added an additional 200 negative genes that showed high overall variations in all experiments to form a 300-gene test set.

Using the default setting in BEST, we identified 57 target genes (Supplementary Table S4) and 139 experimental conditions as foreground. Thirty-three of the target genes (58%) were in the RegulonDB target set (highly significant for enrichment with a p-value of 9.48×10^−13^). A recent microarray analysis suggested that Lrp may affect transcription of as much as 10% of all *E. coli* genes [34]. Therefore it is highly likely that many genes that are not in the RegulonDB target set are indeed regulated by Lrp. Trace plots of the 24 hypothetical Lrp target genes are shown in Supplementary Figures S8, S9, S10, S11, S12, and S13.

We next compared our result to the 43 genes CLR predicted as Lrp targets in [4]. The 239 negative genes we selected actually contain four genes that are on the 43 CLR predicted target gene list but not in the RegulonDB target set. Three of them, metE, ompT and yagU were also identified by BEST as Lrp target genes. In fact, they ranked first, second and sixth in the 24 hypothetical Lrp targets genes listed in Supplementary Table S2. Interestingly, two of them, ompT and yagU have been confirmed to be bound by Lrp *in vivo* using ChIP-qPCR [4]. Furthermore, the scan of Lrp motif indicates that all three genes contain a putative Lrp motif in their intergenic regions upstream.

We also plotted the histograms of expression levels obtained from the top and bottom five experimental conditions sorted by the Bayes ratio comparing whether an experimental condition is foreground or background (Supplementary Figure S14). We again observe strong bi-modal shapes in the histograms representing the top five experimental conditions but not in the histograms representing the bottom five.

### 2.3 Query result from other TFs

In addition to Lrp, we also ran BEST on six other TFs (PdhR, FecI, LexA, FlhC, FlhD and FliA) to test its performance. Among them, LexA, a major regulator of DNA repair, is known to have a single well-conserved DNA binding motif. It is one of the best-perturbed regulators in the microarray compendium due to the compendium's emphasis on DNA-damaging conditions [4]. Other TFs either regulate a large number of genes or have substantial connectivity in the network mapped by the CLR Algorithm [4]. For each TF, we built a test set including all its target genes listed in regulonDB, together with genes predicted by CLR as target. We also included ∼100 genes which displayed the most variation across the 305 experimental conditions as negative signals. The complete results are summarized in the Supplementary Material S1 and all BEST predicted target genes are listed in Supplementary Tables S5, S6, S7, S8, S9, and S10. From these lists, we see that except for PdhR, the majority of target genes listed in regulonDB were identified by BEST. For example, all six FecI target genes, 29 out of 30 FlhC target genes and 41 out of 42 FliA target genes were identified. Furthermore, BEST identified all CLR predicted target genes at 60% precision and numerous additional known target genes.

## Discussion

In summary, we developed a model-based query algorithm based on the Bayesian model selection framework. BEST, a computer program implements this algorithm, is able to query large and heterogeneous microarray gene expression databases for regulon discovery. The query operation considered here can be viewed as a classification procedure where genes sharing similar expression profiles with the query gene belong to one group and the rest belong to the other. Therefore, we considered BEST a supervised learning tool. The key feature of BEST is its ability to recognize co-expression under only a subset of experimental conditions.

In microarray experiments with only a few sample/experimental conditions, the GBA principle has been successfully applied to identify regulons of key TFs [35]. When the experimental conditions are abundant and heterogeneous such as in the case of microarray compendium, the previous strategy will not be as successful since most TFs are only active under certain specific conditions and beyond those conditions no tight correlation is expected between TF and its regulons. BEST is built under the hypothesis that the correlation between TF and its regulon only hold in a subset of conditions. The objective of BEST is to simultaneously predict regulon of a TF and the experimental conditions associated with them. Tests conducted on simulated as well as real datasets indicated that the new algorithm works well and outperforms methods based on global correlation measures, especially when there is substantial noise and moderate distortion in the data.

We are encouraged that when applying BEST to the real *E. coli* compendium data, the majority of genes predicted by BEST as Lrp targets are known target genes of the TF. Interestingly, numerous genes identified show inversed correlation pattern with Lrp. Table 2 lists four such genes, three of them are known to be regulated by Lrp, and the other one showed a very similar pattern with the three known ones. None of these four genes is predicted by CLR. We also believe that many of the “false positive” genes are likely to be real Lrp target genes as well since as many as 10% of all *E. coli* genes are believed to be regulated by Lrp [34] which is significantly larger than the size of the current RegulonDB target set. We also tested major TFs whose target set is larger than ten. Querying these TFs showed that BEST is able to identify the majority of their known target genes. These results suggested that the hypothesis BEST assumed is reasonable. Using microarray compendium data, we are able to generate high confidence and testable hypothesis on TF-regulon relationships.

On the other hand, there are numerous genes in the RegulonDB target sets that were not identified by BEST. Visual inspection of these gene expression trace plots confirms that their expression profiles do not resemble the TF that is supposed to regulate them. This observation suggests that there are limitations on using the GBA principle on gene expression information alone to identify regulons of a TF. There are various reasons why GBA is insufficient to identify the full set of regulon. It is possible that the compendium does not include the experimental conditions under which these genes were regulated by the TF. It is possible that microarray gene expression data is not accurate enough due to measurement error and its limitation in quantifying low-level expression. It is also possible that due to the complexity in regulatory mechanism, some TF-regulon relationships do not imply co-expression under any condition. For example, the TF may require the presence of co-factors or signaling molecules to exert its regulatory function. Other complex regulatory mechanisms such as post-translational modification, chromatin modification, and microRNA regulation may also explain what we observed.

In this study, we assumed that all columns are independent and there is no covariance. This is because replicates in our data have already been merged and adding covariance will significantly increase the complexity of our model. Admittedly, when there are biological or technical replicates, adding covariance in our model will improve the result. We plan to add this option in future releases of BEST.

It is possible to perform a genome-wide search using BEST for genes co-expressed with the query gene. To reduce computation time and to maximize the chance of finding biologically meaningful targets, we recommend a filtering step to reduce the search space. In this study, we adopted a variance filter, which is typical in large-scale gene expression clustering analysis [36] to remove genes that show less variation than the query gene when considering all experimental conditions. We tested this strategy on Lrp in *E. coli*. There are 524 genes (out of 4217 in total, 12%) with total expression variance greater than that of Lrp. They contain 30 genes (out of 61, 49%) that are in the RegulonDB target set. Running BEST with the default setting on this dataset identified 77 genes as targets. Among them, 18 are among the 30 known Lrp target genes (enrichment p-value is 3.32×10^−9^). Compared to the CLR prediction in [4], seven of the 43 CLR predicted target genes that are not in the RegulonDB targets set are among the 524 genes tested. Six of them, gdhA, metE, ompT, pntA, thrA, yagU were also identified by BEST. All but metE have been confirmed *in vivo* as Lrp targets using ChIP-qPCR [4]. The 139 experimental conditions identified by BEST as foreground are essentially the same as in the results from the 100- or 300-gene test sets. These results confirmed the feasibility of our genome-wide search strategy. One can lower the variance threshold to expand the search space if longer computing time can be tolerated.

The statistical model adopted in BEST is closely related to those used in various model-based clustering methods designed for analyzing microarray data [37]–[42]. However, as a supervised learning tool, BEST is able to automatically distinguish the two sets of genes using the expression profile of the pre-specified query gene. This is particularly valuable for searching specific expression patterns of interest. The user can even specify a custom expression pattern to search. In addition, our method allows linearly transformed expression patterns to be recognized and is robust against sporadic outliers in the data.

Our algorithm is built under the Bayesian model selection framework, which may easily incorporate prior biological information. For example, some genes or experimental conditions can be designated as targets or foreground. Similarly, informative priors on cell indicators can help to rule out some sporadic outliers.

MCMC-based methods are typically computation-intensive and therefore time-consuming. BEST's running time depends on the number of iterations and on the size of the dataset. In the study on *E. coli* microarray compendium dataset, using the default setting which is ten parallel chains each with 50 cycles, searching 100 genes takes about 30 minutes on a PowerMac with dual 2.5 GHz processors. Searching 300 and 524 genes takes about 3 hours and 30 hours respectively. A computer program named BEST has been developed based on the aforementioned algorithm. BEST can be downloaded at http://www.sph.umich.edu/csg/qin/BEST.

## Supporting Information

Formula S1 (0.09 MB PDF)

Material S1 (0.10 MB PDF)

Table S1 (0.02 MB DOC)

Table S2 (0.02 MB DOC)

Table S3 (0.04 MB DOC)

Table S4 (0.02 MB DOC)

Table S5 (0.02 MB DOC)

Table S6 (0.02 MB DOC)

Table S7 (0.02 MB DOC)

Table S8 (0.02 MB DOC)

Table S9 (0.02 MB DOC)

Table S10 (0.02 MB DOC)

Figure S1 ROC curves for various query methods when applying to synthetic datasets simulated under different settings and when there are 100% foreground columns. BEST A default setting; BEST B allowing exclusion of individual cells from the foreground; BEST C fixing the indicator variables of five true target genes and five true experimental conditions as 1. A. No linear transformation nor cell-level noise. B. With linear transformation only. C. With cell-level noise only. D. With both linear transformation and cell-level noise.
(0.08 MB TIF)

Figure S2 ROC curves for various query methods when applying to synthetic datasets simulated under different settings and when there are 75% foreground columns. BEST A default setting; BEST B allowing exclusion of individual cells from the foreground; BEST C fixing the indicator variables of five true target genes and five true experimental conditions as 1. A. No linear transformation nor cell-level noise. B. With linear transformation only. C. With cell-level noise only. D. With both linear transformation and cell-level noise.
(0.09 MB TIF)

Figure S3 ROC curves for various query methods when applying to synthetic datasets simulated under different settings and when there are 50% foreground columns. BEST A default setting; BEST B allowing exclusion of individual cells from the foreground; BEST C fixing the indicator variables of five true target genes and five true experimental conditions as 1. A. No linear transformation nor cell-level noise. B. With linear transformation only. C. With cell-level noise only. D. With both linear transformation and cell-level noise.
(0.08 MB TIF)

Figure S4 Log-likelihood trace plots of the ten parallel chains resulted from the BEST run on 100-gene and 300-gene test sets selected from the E. coli microarray compendium.
(0.06 MB TIF)

Figure S5 Sequence logo plot [1] and position specific weight matrix (PSWM) for the motif of transcription factor Lrp. Lrp motif is downloaded from regulonDB: http://regulondb.ccg.unam.mx/data/Matrix_AlignmentSet.txt. The logo plot was generated by the seqLogo program [4].
(0.03 MB TIF)

Figure S6 Boxplots of Pearson correlation coefficients. A. Boxplots of Pearson correlations between expression profiles of the 61 experimentally verified Lrp target genes and Lrp. The left one summarize correlations measured in the 162 background experiments and the right one summarize correlations measured in the 143 foreground experiments. A paired t-test comparing the two sets of correlation coefficients returns a p-value of 0.0079. B. Boxplots of Pearson correlations between expression profiles of the 28 genes BEST indentified as Lrp target. The left one summarize correlations measured in the 162 background experiments and the right one summarize correlations measured in the 143 foreground experiments. A paired t-test comparing the two sets of correlation coefficients returns a p-value of 1.948×10–12.
(0.08 MB TIF)

Figure S7 A. Histograms of expression profile differences (zij) in the top five experimental conditions (foreground). B. Histogram of expression profile differences (zij) in the bottom five experimental conditions (background). Data used here is the 100-gene test set selected from the E. coli microarray compendium.
(0.08 MB TIF)

Figure S8 Trace plots of 24 predicted Lrp target genes identified by BEST that are not in the RegulonDB target set (Part 1). Black lines indicate the query gene-Lrp, the red line indicate the potential target genes. Only the 139 foreground experimental conditions identified by BEST were shown in these plots
(0.07 MB TIF)

Figure S9 Trace plots of 24 predicted Lrp target genes identified by BEST that are not in the RegulonDB target set (Part 2). Black lines indicate the query gene-Lrp, the red line indicate the potential target genes. Only the 139 foreground experimental conditions identified by BEST were shown in these plots
(0.08 MB TIF)

Figure S10 Trace plots of 24 predicted Lrp target genes identified by BEST that are not in the RegulonDB target set (Part 3). Black lines indicate the query gene-Lrp, the red line indicate the potential target genes. Only the 139 foreground experimental conditions identified by BEST were shown in these plots
(0.08 MB TIF)

Figure S11 Trace plots of 24 predicted Lrp target genes identified by BEST that are not in the RegulonDB target set (Part 4). Black lines indicate the query gene-Lrp, the red line indicate the potential target genes. Only the 139 foreground experimental conditions identified by BEST were shown in these plots
(0.08 MB TIF)

Figure S12 Trace plots of 24 predicted Lrp target genes identified by BEST that are not in the RegulonDB target set (Part 5). Black lines indicate the query gene-Lrp, the red line indicate the potential target genes. Only the 139 foreground experimental conditions identified by BEST were shown in these plots
(0.08 MB TIF)

Figure S13 Trace plots of 24 predicted Lrp target genes identified by BEST that are not in the RegulonDB target set (Part 6). Black lines indicate the query gene-Lrp, the red line indicate the potential target genes. Only the 139 foreground experimental conditions identified by BEST were shown in these plots
(0.07 MB TIF)

Figure S14 A. Histograms of expression profile differences (zij) in the top five experimental conditions (foreground). B. Histogram of expression profile differences (zij) in the bottom five experimental conditions (background). Data used here is the 300-gene test set selected from the E. coli microarray compendium
(0.08 MB TIF)

## References

[1] Schena M, Shalon D, Davis RW, Brown PO (1995). Quantitative monitoring of gene expression patterns with a complementary DNA microarray.. Science.

[2] Lockhart DJ, Dong H, Byrne MC, Follettie MT, Gallo MV (1996). Expression monitoring by hybridization to high-density oligonucleotide arrays.. Nat Biotechnol.

[3] Brown PO, Botstein D (1999). Exploring the new world of the genome with DNA microarrays.. Nat Genet.

[4] Faith JJ, Hayete B, Thaden JT, Mogno I, Wierzbowski J (2007). Large-scale mapping and validation of Escherichia coli transcriptional regulation from a compendium of expression profiles.. PLoS Biol.

[5] Hughes TR, Marton MJ, Jones AR, Roberts CJ, Stoughton R (2000). Functional discovery via a compendium of expression profiles.. Cell.

[6] Kim SK, Lund J, Kiraly M, Duke K, Jiang M (2001). A gene expression map for Caenorhabditis elegans.. Science.

[7] Hibbs MA, Hess DC, Myers CL, Huttenhower C, Li K (2007). Exploring the functional landscape of gene expression: directed search of large microarray compendia.. Bioinformatics.

[8] Eisen MB, Spellman PT, Brown PO, Botstein D (1998). Cluster analysis and display of genome-wide expression patterns.. Proc Natl Acad Sci U S A.

[9] Bassett DE, Eisen MB, Boguski MS (1999). Gene expression informatics–it's all in your mine.. Nat Genet.

[10] Walker MG, Volkmuth W, Sprinzak E, Hodgson D, Klingler T (1999). Prediction of gene function by genome-scale expression analysis: prostate cancer-associated genes.. Genome Res.

[11] Quackenbush J (2003). Genomics. Microarrays–guilt by association.. Science.

[12] Wolfe CJ, Kohane IS, Butte AJ (2005). Systematic survey reveals general applicability of “guilt-by-association” within gene coexpression networks.. BMC Bioinformatics.

[13] Mootha VK, Lepage P, Miller K, Bunkenborg J, Reich M (2003). Identification of a gene causing human cytochrome c oxidase deficiency by integrative genomics.. Proc Natl Acad Sci U S A.

[14] Butte AJ, Kohane IS (2000). Mutual information relevance networks: functional genomic clustering using pairwise entropy measurements.. Pac Symp Biocomput.

[15] Margolin AA, Nemenman I, Basso K, Wiggins C, Stolovitzky G (2006). ARACNE: an algorithm for the reconstruction of gene regulatory networks in a mammalian cellular context.. BMC Bioinformatics.

[16] Neuwald AF, Kannan N, Poleksic A, Hata N, Liu JS (2003). Ran's C-terminal, Basic Patch, and Nucleotide Exchange Mechanisms in Light of a Canonical Structure for Rab, Rho, Ras, and Ran GTPases.. Genome Res.

[17] Tanay A, Sharan R, Shamir R (2002). Discovering statistically significant biclusters in gene expression data.. Bioinformatics.

[18] Cheng Y, Church GM (2000). Biclustering of expression data.. Proc Int Conf Intell Syst Mol Biol.

[19] Getz G, Levine E, Domany E (2000). Coupled two-way clustering analysis of gene microarray data.. Proc Natl Acad Sci U S A.

[20] Sheng Q, Moreau Y, De Moor B (2003). Biclustering microarray data by Gibbs sampling.. Bioinformatics.

[21] Madeira SC, Oliveira AL (2004). Biclustering algorithms for biological data analysis: a survey.. IEEE/ACM Trans Comput Biol Bioinform.

[22] Qian J, Dolled-Filhart M, Lin J, Yu H, Gerstein M (2001). Beyond synexpression relationships: local clustering of time-shifted and inverted gene expression profiles identifies new, biologically relevant interactions.. J Mol Biol.

[23] Smith TF, Waterman MS (1981). Identification of common molecular subsequences.. J Mol Biol.

[24] Dhollander T, Sheng Q, Lemmens K, Moor BD, Marchal K (2007). Query-driven module discovery in microarray data.. Bioinformatics.

[25] Owen AB, Stuart J, Mach K, Villeneuve AM, Kim S (2003). A gene recommender algorithm to identify coexpressed genes in C. elegans.. Genome Res.

[26] Gelman A, Carlin JB, Stern HS, Rubin DB (1995). Bayesian data analysis.

[27] Chen R, Liu JS (1996). Predictive Updating Methods With Application to Bayesian Classification.. Journal of the Royal Statistical Society Series B-Methodological.

[28] Gelfand AE, Smith AFM (1990). Sampling-based approaches to calculating marginal densities.. Journal of the American Statistical Association.

[29] Liu JS (2001). Monte Carlo Strategies in Scientific Computing.

[30] Faith JJ, Driscoll ME, Fusaro VA, Cosgrove EJ, Hayete B (2008). Many Microbe Microarrays Database: uniformly normalized Affymetrix compendia with structured experimental metadata.. Nucleic Acids Res.

[31] Brinkman AB, Ettema TJ, de Vos WM, van der Oost J (2003). The Lrp family of transcriptional regulators.. Mol Microbiol.

[32] Salgado H, Gama-Castro S, Peralta-Gil M, Diaz-Peredo E, Sanchez-Solano F (2006). RegulonDB (version 5.0): Escherichia coli K-12 transcriptional regulatory network, operon organization, and growth conditions.. Nucleic Acids Res.

[33] Urbanowski ML, Stauffer LT, Stauffer GV (2000). The gcvB gene encodes a small untranslated RNA involved in expression of the dipeptide and oligopeptide transport systems in Escherichia coli.. Mol Microbiol.

[34] Tani TH, Khodursky A, Blumenthal RM, Brown PO, Matthews RG (2002). Adaptation to famine: a family of stationary-phase genes revealed by microarray analysis.. Proc Natl Acad Sci U S A.

[35] Roth FP, Hughes JD, Estep PW, Church GM (1998). Finding DNA regulatory motifs within unaligned noncoding sequences clustered by whole-genome mRNA quantitation.. Nature Biotechnology.

[36] Tamayo P, Slonim D, Mesirov J, Zhu Q, Kitareewan S (1999). Interpreting patterns of gene expression with self-organizing maps: methods and application to hematopoietic differentiation.. Proc Natl Acad Sci U S A.

[37] McLachlan GJ, Bean RW, Peel D (2002). A mixture model-based approach to the clustering of microarray expression data.. Bioinformatics.

[38] Yeung KY, Ruzzo WL (2001). Principal component analysis for clustering gene expression data.. Bioinformatics.

[39] Ghosh D, Chinnaiyan AM (2002). Mixture modelling of gene expression data from microarray experiments.. Bioinformatics.

[40] Medvedovic M, Sivaganesan S (2002). Bayesian infinite mixture model based clustering of gene expression profiles.. Bioinformatics.

[41] Qin ZS (2006). Clustering microarray gene expression data using weighted Chinese restaurant process.. Bioinformatics.

[42] Kim S, Tadesse MG, Vannucci M (2006). Variable selection in clustering via Dirichlet process mixture models.. Biometrika.

