# Supplementary material for: Query Large Scale Microarray Compendium Datasets Using a Model-Based Bayesian Approach with Variable Selection
Source: PLoS One. 2009 Feb 13;4(2):e4495. doi: 10.1371/journal.pone.0004495 (PMC2637418; doi:10.1371/journal.pone.0004495)
Supplement: Formula S1 — (0.09 MB PDF) [file pone.0004495.s001.pdf]

Supplementary material

1 Formulas

The overall likelihood can be expressed as:

$$P(Z|R,E,\Theta)=\prod_{i=1}^{N}\prod_{j=1}^{M}\left[\left(\frac{1}{\sqrt{2\pi}\sigma_{1j}}\ e^{-\frac{z_{ij}^2}{2\sigma_{1j}^2}}\right)^{r_i\cdot e_j}\cdot\left(\frac{1}{\sqrt{2\pi}\sigma_{0j}}\ e^{-\frac{(z_{ij}-\mu_{0j})^2}{2\sigma_{0j}^2}}\right)^{1-r_i\cdot e_j}\right]$$

$$\begin{gathered} p(\sigma_{1j}^2)\sim Inverse\ Gamma(\alpha_{1j},\beta_{1j}) \\ p(\sigma_{0j}^2)\sim Inverse\ Gamma(\alpha_{0j},\beta_{0j}) \\ p(\mu_{0j}|\sigma_{0j}^2)\sim N(\tau_{0j},\sigma_{0j}^2) \\ p(r_i)\sim Bernoulli(\pi_{r_i}) \\ p(e_j)\sim Bernoulli(\pi_{e_j}) \\ i=1,\cdots,N \qquad j=1,\cdots,M \end{gathered}$$

$$\begin{aligned} p(R,E,\Theta|Z) &\propto p(R,E,\Theta,Z) \\ &= p(Z|R,E,\Theta)\prod_{i=1}^{N}p(r_i)\prod_{j=1}^{M}\left[p(\mu_{0j}|\sigma_{0j}^2)p(\sigma_{0j}^2)p(\sigma_{1j}^2)p(e_j)\right] \end{aligned}$$

The marginal posterior distribution is:

$$\begin{aligned} p(R,E|Z) &= \int p(R,E,\Theta|Z)\ d\Theta \\ &\propto \int p(Z|R,E,\Theta)\prod_{i=1}^{N}p(r_i)\prod_{j=1}^{M}\left[p(\mu_{0j}|\sigma_{0j}^2)p(\sigma_{0j}^2)p(\sigma_{1j}^2)p(e_j)\right]\ d\Theta \\ &\propto \int\prod_{i=1}^{N}\prod_{j=1}^{M}\left[\left(\sigma_{1j}^{-1}e^{-\frac{z_{ij}^2}{2\sigma_{1j}^2}}\right)^{r_i\cdot e_j}\cdot\left(\sigma_{0j}^{-1}e^{-\frac{(z_{ij}-\mu_{0j})^2}{2\sigma_{0j}^2}}\right)^{1-r_i\cdot e_j}\right]\prod_{i=1}^{N}\pi_{r_i}^{r_i}(1-\pi_{r_i})^{1-r_i} \\ &\quad \prod_{j=1}^{M}\left[\sigma_{0j}^{-1}e^{-\frac{(\mu_{0j}-\tau_{0j})^2}{2\sigma_{0j}^2}}\cdot(\sigma_{0j}^2)^{-(\alpha_{0j}+1)}e^{-\frac{\beta_{0j}}{\sigma_{0j}^2}}(\sigma_{1j}^2)^{-(\alpha_{1j}+1)}e^{-\frac{\beta_{1j}}{\sigma_{1j}^2}}\pi_{e_j}^{e_j}(1-\pi_{e_j})^{1-e_j}\right]\ d\Theta \end{aligned}$$

Divide the integral into two part: I_1 and I_2:

$$
\begin{aligned}
I_1 &= \int \prod_{i=1}^{N}\prod_{j=1}^{M}\left(\sigma_{1j}^{-1}\exp\left\{-\frac{z_{ij}^2}{2\sigma_{1j}^2}\right\}\right)^{r_i\cdot e_j} \cdot \prod_{j=1}^{M}(\sigma_{1j}^2)^{-(\alpha_{1j}+1)}e^{-\frac{\beta_{1j}}{\sigma_{1j}^2}}\ d\sigma_{1j}^2 \\
&= \prod_{j=1}^{M}\int\prod_{i=1}^{N}\left(\sigma_{1j}^{-1}\exp\left\{-\frac{z_{ij}^2}{2\sigma_{1j}^2}\right\}\right)^{r_i\cdot e_j} \cdot (\sigma_{1j}^2)^{-(\alpha_{1j}+1)}e^{-\frac{\beta_{1j}}{\sigma_{1j}^2}}\ d\sigma_{1j}^2 \\
&= \prod_{j=1}^{M}\int \sigma_{1j}^{-e_j\sum_{i=1}^N r_i}\exp\left\{-\frac{e_j}{2\sigma_{1j}^2}\sum_{i=1}^{N}z_{ij}^2 r_i\right\}\cdot\ (\sigma_{1j}^2)^{-(\alpha_{1j}+1)}e^{-\frac{\beta_{1j}}{\sigma_{1j}^2}}\ d\sigma_{1j}^2 \\
&= \prod_{j=1}^{M}\int(\sigma_{1j}^2)^{-(\alpha_{1j}+1+\frac{e_j}{2}\sum_{i=1}^N r_i)}\exp\left\{-\frac{1}{\sigma_{1j}^2}\left(\beta_{1j}+\frac{e_j}{2}\sum_{i=1}^{N}z_{ij}^2 r_i\right)\right\}\ d\sigma_{1j}^2 \\
&= \prod_{j=1}^{M}\frac{\Gamma\left(\alpha_{1j}+\frac{e_j}{2}\sum_{i=1}^N r_i\right)}{\left[\beta_{1j}+\frac{e_j}{2}\sum_{i=1}^N z_{ij}^2 r_i\right]^{\alpha_{1j}+\frac{e_j}{2}\sum_{i=1}^N r_i}}
\end{aligned}
$$

Let $f_{ij} = 1 - r_i e_j$, $A_j = \sum_{i=1}^N z_{ij}^2 f_{ij}$, $B_j = \sum_{i=1}^N z_{ij} f_{ij}$, $C_j = \sum_{i=1}^N f_{ij}$.

$$
\begin{aligned}
I_2 &= \int\int\prod_{i=1}^{N}\prod_{j=1}^{M}\left(\sigma_{0j}^{-1}e^{-\frac{(z_{ij}-\mu_{0j})^2}{2\sigma_{0j}^2}}\right)^{1-r_i\cdot e_j}\sigma_{0j}^{-1}e^{-\frac{(\mu_{0j}-\tau_{0j})^2}{2\sigma_{0j}^2}}(\sigma_{0j}^2)^{-(\alpha_{0j}+1)}e^{-\frac{\beta_{0j}}{\sigma_{0j}^2}}\ d\mu_{0j}\ d\sigma_{0j}^2 \\
&= \prod_{j=1}^{M}\int\int(\sigma_{0j}^2)^{-\frac{C_j+3}{2}-\alpha_{0j}}\exp\left\{-\frac{1}{2\sigma_{0j}^2}\left(\sum_{i=1}^{N}(z_{ij}-\mu_{0j})^2 f_{ij}+(\mu_{0j}-\tau_{0j})^2+2\beta_{0j}\right)\right\}\ d\mu_{0j}\ d\sigma_{0j}^2 \\
&= \prod_{j=1}^{M}\int\int(\sigma_{0j}^2)^{-\frac{C_j+3}{2}-\alpha_{0j}} \\
&\quad \exp\left\{-\frac{1}{2\sigma_{0j}^2}\left[A_j+\tau_{0j}^2+2\beta_{0j}-2\mu_{0j}\left(B_j+\tau_{0j}\right)+\mu_{0j}^2(1+C_j)\right]\right\}\ d\mu_{0j}\ d\sigma_{0j}^2 \\
&= \prod_{j=1}^{M}\int\left\{\int(\sigma_{0j}^2)^{-\frac{C_j+3}{2}-\alpha_{0j}}\exp\left[-\frac{1+C_j}{2\sigma_{0j}^2}\left(\mu_{0j}-\frac{B_j+\tau_{0j}}{1+C_j}\right)^2\right]\ d\mu_{0j}\right\} \\
&\quad \exp\left\{-\frac{1}{2\sigma_{0j}^2}\left[A_j+\tau_{0j}^2+2\beta_{0j}-\frac{(B_j+\tau_{0j})^2}{1+C_j}\right]\right\}d\sigma_{0j}^2 \\
&\propto \prod_{j=1}^{M}(1+C_j)^{-1/2}\int(\sigma_{0j}^2)^{-\frac{C_j+2}{2}-\alpha_{0j}}\exp\left\{-\frac{1}{2\sigma_{0j}^2}\left[A_j+\tau_{0j}^2+2\beta_{0j}-\frac{(B_j+\tau_{0j})^2}{1+C_j}\right]\right\}d\sigma_{0j}^2 \\
&= \prod_{j=1}^{M}\frac{(1+C_j)^{-1/2}\Gamma(\alpha_{0j}+C_j/2)2^{\alpha_{0j}+C_j/2}}{\left[A_j+\tau_{0j}^2+2\beta_{0j}-\frac{(B_j+\tau_{0j})^2}{1+C_j}\right]^{\alpha_{0j}+C_j/2}}
\end{aligned}
$$

Then the log transformed marginal posterior distribution is:

$$
\begin{aligned}
\log p(R,E|Z) &= \log I_1 + \log I_2 + \sum_{i=1}^{N} r_i \log \frac{\pi_{r_i}}{1-\pi_{r_i}} + \sum_{j=1}^{M} e_j \log \frac{\pi_{e_j}}{1-\pi_{e_j}} + constant \\
&= \sum_{j=1}^{M} \log\Gamma\left(\alpha_{1j} + \frac{e_j}{2}\sum_{i=1}^{N} r_i\right) - \sum_{j=1}^{M}\left(\alpha_{1j} + \frac{e_j}{2}\sum_{i=1}^{N} r_i\right)\log\left(\beta_{1j} + \frac{e_j}{2}\sum_{i=1}^{N} z_{ij}^2 r_i\right) \\
&- \sum_{j=1}^{M}\frac{1}{2}\log(1+C_j) + \sum_{j=1}^{M}\log\Gamma(\alpha_{0j} + C_j/2) \\
&- \sum_{j=1}^{M}(\alpha_{0j} + C_j/2)\log\left[A_j/2 + \tau_{0j}^2/2 + \beta_{0j} - \frac{(B_j+\tau_{0j})^2}{2(1+C_j)}\right] \\
&+ \sum_{i=1}^{N} r_i \log \frac{\pi_{r_i}}{1-\pi_{r_i}} + \sum_{j=1}^{M} e_j \log \frac{\pi_{e_j}}{1-\pi_{e_j}} + constant \\
&= \sum_{j=1}^{M} \log\Gamma\left(\alpha_{1j} + \frac{e_j}{2}\sum_{i=1}^{N} r_i\right) - \sum_{j=1}^{M}\left(\alpha_{1j} + \frac{e_j}{2}\sum_{i=1}^{N} r_i\right)\log\left(\beta_{1j} + \frac{e_j}{2}\sum_{i=1}^{N} z_{ij}^2 r_i\right) \\
&- \sum_{j=1}^{M}\frac{1}{2}\log\left[1+\sum_{i=1}^{N}(1-r_ie_j)\right] + \sum_{j=1}^{M}\log\Gamma\left(\alpha_{0j} + \sum_{i=1}^{N}\frac{1-r_ie_j}{2}\right) \\
&- \sum_{j=1}^{M}\left(\alpha_{0j} + \sum_{i=1}^{N}\frac{1-r_ie_j}{2}\right) \\
&\times \log\left\{\frac{\tau_{0j}^2}{2} + \beta_{0j} + \sum_{i=1}^{N}\frac{z_{ij}^2(1-r_ie_j)}{2} - \frac{\left(\sum_{i=1}^{N} z_{ij}(1-r_ie_j)+\tau_{0j}\right)^2}{2\left[1+\sum_{i=1}^{N}(1-r_ie_j)\right]}\right\} \\
&+ \sum_{i=1}^{N} r_i \log \frac{\pi_{r_i}}{1-\pi_{r_i}} + \sum_{j=1}^{M} e_j \log \frac{\pi_{e_j}}{1-\pi_{e_j}} + constant \qquad \text{(S1)}
\end{aligned}
$$

Allowing cell level noise, the overall likelihood is modified as follows:

$$P(Z|R,E,C,\Theta) = \prod_{i=1}^{N}\prod_{j=1}^{M}\left[\left(\frac{1}{\sqrt{2\pi}\sigma_{1j}}\ e^{-\frac{z_{ij}^2}{2\sigma_{1j}^2}}\right)^{r_i\cdot e_j\cdot c_{ij}} \cdot \left(\frac{1}{\sqrt{2\pi}\sigma_{0j}}\ e^{-\frac{(z_{ij}-\mu_{0j})^2}{2\sigma_{0j}^2}}\right)^{1-r_i\cdot e_j\cdot c_{ij}}\right]$$

$$p(\sigma_{1j}^2) \sim Inverse\ Gamma(\alpha_{1j},\beta_{1j})$$
$$p(\sigma_{0j}^2) \sim Inverse\ Gamma(\alpha_{0j},\beta_{0j})$$
$$p(\mu_{0j}|\sigma_{0j}^2) \sim N(\tau_{0j},\sigma_{0j}^2)$$
$$p(r_i) \sim Bernoulli(\pi_{r_i})$$
$$p(e_j) \sim Bernoulli(\pi_{e_j})$$
$$p(c_{ij}) \sim Bernoulli(\pi_{c_{ij}})$$
$$i = 1,\cdots,N \qquad j = 1,\cdots,M$$

$$\begin{aligned}
p(R,E,C,\Theta|Z) &\propto p(R,E,C,\Theta,Z) \\
&= p(Z|R,E,C,\Theta)\prod_{i=1}^{N}p(r_i)\prod_{j=1}^{M}\left[p(\mu_{0j}|\sigma_{0j}^2)p(\sigma_{0j}^2)p(\sigma_{1j}^2)p(e_j)\right]\prod_{i=1}^{N}\prod_{j=1}^{M}p(c_{ij})
\end{aligned}$$

The marginal posterior distribution is:

$$\begin{aligned}
p(R,E,C|Z) &= \int p(R,E,C,\Theta|Z)\ d\Theta \\
&\propto \int p(Z|R,E,C,\Theta)\prod_{j=1}^{M}p(\mu_{0j}|\sigma_{0j}^2)\prod_{j=1}^{M}p(\sigma_{0j}^2)\prod_{j=1}^{M}p(\sigma_{1j}^2) \\
&\cdot \prod_{i=1}^{N}p(r_i)\prod_{j=1}^{M}p(e_j)\prod_{i=1}^{N}\prod_{j=1}^{M}p(c_{ij})\ d\Theta \\
&\propto \int\prod_{i=1}^{N}\prod_{j=1}^{M}\left[\left(\sigma_{1j}^{-1}e^{-\frac{z_{ij}^2}{2\sigma_{1j}^2}}\right)^{r_i\cdot e_j\cdot c_{ij}} \cdot \left(\sigma_{0j}^{-1}e^{-\frac{(z_{ij}-\mu_{0j})^2}{2\sigma_{0j}^2}}\right)^{1-r_i\cdot e_j\cdot c_{ij}}\right] \\
&\quad \cdot\prod_{j=1}^{M}\sigma_{0j}^{-1}e^{-\frac{(\mu_{0j}-\tau_{0j})^2}{2\sigma_{0j}^2}} \cdot\prod_{j=1}^{M}(\sigma_{0j}^2)^{-(\alpha_{0j}+1)}e^{-\frac{\beta_{0j}}{\sigma_{0j}^2}} \cdot\prod_{j=1}^{M}(\sigma_{1j}^2)^{-(\alpha_{1j}+1)}e^{-\frac{\beta_{1j}}{\sigma_{1j}^2}} \\
&\quad \prod_{i=1}^{N}\pi_{r_i}^{r_i}(1-\pi_{r_i})^{1-r_i}\prod_{j=1}^{M}\pi_{e_j}^{e_j}(1-\pi_{e_j})^{1-e_j}\prod_{i=1}^{N}\prod_{j=1}^{M}\pi_{c_{ij}}^{c_{ij}}(1-\pi_{c_{ij}})^{1-c_{ij}}\ d\Theta
\end{aligned}$$

Divide the integral into two part: I_1 and I_2:

$$\begin{aligned}
I_1 &= \int \prod_{i=1}^{N}\prod_{j=1}^{M}\left(\sigma_{1j}^{-1}\exp\left\{-\frac{z_{ij}^2}{2\sigma_{1j}^2}\right\}\right)^{r_i\cdot e_j\cdot c_{ij}} \cdot \prod_{j=1}^{M}(\sigma_{1j}^2)^{-(\alpha_{1j}+1)}e^{-\frac{\beta_{1j}}{\sigma_{1j}^2}}\ d\sigma_{1j}^2 \\
&= \prod_{j=1}^{M}\int \prod_{i=1}^{N}\left(\sigma_{1j}^{-1}\exp\left\{-\frac{z_{ij}^2}{2\sigma_{1j}^2}\right\}\right)^{r_i\cdot e_j\cdot c_{ij}} \cdot (\sigma_{1j}^2)^{-(\alpha_{1j}+1)}e^{-\frac{\beta_{1j}}{\sigma_{1j}^2}}\ d\sigma_{1j}^2 \\
&= \prod_{j=1}^{M}\int \sigma_{1j}^{-e_j\sum_{i=1}^{N} r_i c_{ij}}\exp\left\{-\frac{e_j}{2\sigma_{1j}^2}\sum_{i=1}^{N} z_{ij}^2 r_i c_{ij}\right\}\cdot\ (\sigma_{1j}^2)^{-(\alpha_{1j}+1)}e^{-\frac{\beta_{1j}}{\sigma_{1j}^2}}\ d\sigma_{1j}^2 \\
&= \prod_{j=1}^{M}\int (\sigma_{1j}^2)^{-(\alpha_{1j}+1+\frac{e_j}{2}\sum_{i=1}^{N} r_i c_{ij})}\exp\left\{-\frac{1}{\sigma_{1j}^2}\left(\beta_{1j}+\frac{e_j}{2}\sum_{i=1}^{N} z_{ij}^2 r_i c_{ij}\right)\right\}\ d\sigma_{1j}^2 \\
&= \prod_{j=1}^{M}\frac{\Gamma\left(\alpha_{1j}+\frac{e_j}{2}\sum_{i=1}^{N} r_i c_{ij}\right)}{\left[\beta_{1j}+\frac{e_j}{2}\sum_{i=1}^{N} z_{ij}^2 r_i c_{ij}\right]^{\alpha_{1j}+\frac{e_j}{2}\sum_{i=1}^{N} r_i c_{ij}}}
\end{aligned}$$

Let $g_{ij} = 1 - r_i e_j c_{ij}$, $D_j = \sum_{i=1}^{N} z_{ij}^2 g_{ij}$, $E_j = \sum_{i=1}^{N} z_{ij} g_{ij}$, $F_j = \sum_{i=1}^{N} g_{ij}$.

$$\begin{aligned}
I_2 &= \int\int \prod_{i=1}^{N}\prod_{j=1}^{M}\left(\sigma_{0j}^{-1}e^{-\frac{(z_{ij}-\mu_{0j})^2}{2\sigma_{0j}^2}}\right)^{1-r_i\cdot e_j\cdot c_{ij}} \sigma_{0j}^{-1}e^{-\frac{(\mu_{0j}-\tau_{0j})^2}{2\sigma_{0j}^2}}(\sigma_{0j}^2)^{-(\alpha_{0j}+1)}e^{-\frac{\beta_{0j}}{\sigma_{0j}^2}}\ d\mu_{0j}\ d\sigma_{0j}^2 \\
&= \prod_{j=1}^{M}\int\int(\sigma_{0j}^2)^{-\frac{F_j+3}{2}-\alpha_{0j}}\exp\left\{-\frac{1}{2\sigma_{0j}^2}\left(\sum_{i=1}^{N}(z_{ij}-\mu_{0j})^2 g_{ij}+(\mu_{0j}-\tau_{0j})^2+2\beta_{0j}\right)\right\}\ d\mu_{0j}\ d\sigma_{0j}^2 \\
&= \prod_{j=1}^{M}\int\int(\sigma_{0j}^2)^{-\frac{F_j+3}{2}-\alpha_{0j}} \\
&\quad \exp\left\{-\frac{1}{2\sigma_{0j}^2}\left[D_j+\tau_{0j}^2+2\beta_{0j}-2\mu_{0j}\left(E_j+\tau_{0j}\right)+\mu_{0j}^2(1+F_j)\right]\right\}\ d\mu_{0j}\ d\sigma_{0j}^2 \\
&= \prod_{j=1}^{M}\int\left\{\int(\sigma_{0j}^2)^{-\frac{F_j+3}{2}-\alpha_{0j}}\exp\left[-\frac{1+F_j}{2\sigma_{0j}^2}\left(\mu_{0j}-\frac{E_j+\tau_{0j}}{1+F_j}\right)^2\right]\ d\mu_{0j}\right\} \\
&\quad \exp\left\{-\frac{1}{2\sigma_{0j}^2}\left[D_j+\tau_{0j}^2+2\beta_{0j}-\frac{(E_j+\tau_{0j})^2}{1+F_j}\right]\right\}d\sigma_{0j}^2 \\
&\propto \prod_{j=1}^{M}(1+F_j)^{-1/2}\int(\sigma_{0j}^2)^{-\frac{F_j+2}{2}-\alpha_{0j}}\exp\left\{-\frac{1}{2\sigma_{0j}^2}\left[D_j+\tau_{0j}^2+2\beta_{0j}-\frac{(E_j+\tau_{0j})^2}{1+F_j}\right]\right\}d\sigma_{0j}^2 \\
&= \prod_{j=1}^{M}\frac{(1+F_j)^{-1/2}\Gamma(\alpha_{0j}+F_j/2)2^{\alpha_{0j}+F_j/2}}{\left[D_j+\tau_{0j}^2+2\beta_{0j}-\frac{(E_j+\tau_{0j})^2}{1+F_j}\right]^{\alpha_{0j}+F_j/2}}
\end{aligned}$$

Then the log transformed marginal posterior distribution is:

$$\begin{aligned}
\log p(R,E,C|Z) &= \log I_1 + \log I_2 + \sum_{i=1}^{N} r_i \log \frac{\pi_{r_i}}{1-\pi_{r_i}} + \sum_{j=1}^{M} e_j \log \frac{\pi_{e_j}}{1-\pi_{e_j}} \\
&+ \sum_{i=1}^{N}\sum_{j=1}^{M} c_{ij} \log \frac{\pi_{c_{ij}}}{1-\pi_{c_{ij}}} + constant \\
&= \sum_{j=1}^{M} \log\Gamma\left(\alpha_{1j} + \frac{e_j}{2}\sum_{i=1}^{N} r_i c_{ij}\right) \\
&- \sum_{j=1}^{M}\left(\alpha_{1j} + \frac{e_j}{2}\sum_{i=1}^{N} r_i c_{ij}\right)\log\left(\beta_{1j} + \frac{e_j}{2}\sum_{i=1}^{N} z_{ij}^2 r_i c_{ij}\right) \\
&- \sum_{j=1}^{M}\frac{1}{2}\log(1+F_j) + \sum_{j=1}^{M}\log\Gamma(\alpha_{0j} + F_j/2) \\
&- \sum_{j=1}^{M}(\alpha_{0j} + F_j/2)\log\left[D_j/2 + \tau_{0j}^2/2 + \beta_{0j} - \frac{(E_j+\tau_{0j})^2}{2(1+F_j)}\right] \\
&+ \sum_{i=1}^{N} r_i \log \frac{\pi_{r_i}}{1-\pi_{r_i}} + \sum_{j=1}^{M} e_j \log \frac{\pi_{e_j}}{1-\pi_{e_j}} + \sum_{i=1}^{N}\sum_{j=1}^{M} c_{ij} \log \frac{\pi_{c_{ij}}}{1-\pi_{c_{ij}}} + constant \\
&= \sum_{j=1}^{M} \log\Gamma\left(\alpha_{1j} + \frac{e_j}{2}\sum_{i=1}^{N} r_i c_{ij}\right) \\
&- \sum_{j=1}^{M}\left(\alpha_{1j} + \frac{e_j}{2}\sum_{i=1}^{N} r_i c_{ij}\right)\log\left(\beta_{1j} + \frac{e_j}{2}\sum_{i=1}^{N} z_{ij}^2 r_i c_{ij}\right) \\
&- \sum_{j=1}^{M}\frac{1}{2}\log\left[1 + \sum_{i=1}^{N}(1 - r_i e_j c_{ij})\right] + \sum_{j=1}^{M}\log\Gamma\left(\alpha_{0j} + \sum_{i=1}^{N}\frac{1 - r_i e_j c_{ij}}{2}\right) \\
&- \sum_{j=1}^{M}\left(\alpha_{0j} + \sum_{i=1}^{N}\frac{1 - r_i e_j c_{ij}}{2}\right) \\
&\times \log\left\{\frac{\tau_{0j}^2}{2} + \beta_{0j} + \sum_{i=1}^{N}\frac{z_{ij}^2(1 - r_i e_j c_{ij})}{2} - \frac{\left(\sum_{i=1}^{N} z_{ij}(1 - r_i e_j c_{ij}) + \tau_{0j}\right)^2}{2\left[1 + \sum_{i=1}^{N}(1 - r_i e_j c_{ij})\right]}\right\} \\
&+ \sum_{i=1}^{N} r_i \log \frac{\pi_{r_i}}{1-\pi_{r_i}} + \sum_{j=1}^{M} e_j \log \frac{\pi_{e_j}}{1-\pi_{e_j}} + \sum_{i=1}^{N}\sum_{j=1}^{M} c_{ij} \log \frac{\pi_{c_{ij}}}{1-\pi_{c_{ij}}} \\
&+ constant \qquad \text{(S2)}
\end{aligned}$$

2 Prior Specification

We adopt standard conjugate priors for the model parameters. The prior parameters τ_{0j}, α_{0j}, β_{0j}, α_{1j} and β_{1j} are specified as follows:

First we estimate the linear transformation factor $\hat{a}_i$ using least square without intercept. To keep our model simple and avoid over-fitting, the linear transformation factor $\hat{a}_i$ is restricted to be significantly different from 0 and 1. The difference z_{ij} is defined as $y_{ij} - \hat{a}_i \cdot x_j$.

We calculate the sample mean $\hat{\mu}_{0j}$ and sample variance $\hat{v}_{0j}^2$ of the difference z_{ij} in each column, and set $\tau_{0j} = \hat{\mu}_{0j}$. Next we calculate the sample variance $\hat{\sigma}_0^2$ of column variance $\hat{v}_{0j}^2$, and choose α_{0j}, β_{0j}, such that the prior distribution Inverse Gamma$(\alpha_{0j}, \beta_{0j})$ has mean $\hat{v}_{0j}$ and variance $\hat{\sigma}_0^2$.

To choose α_{1j}, β_{1j} in each column, we select z_{ij} with absolute value less than $\hat{\sigma}_{0j}/2$ in that column and calculate their sample variance $\hat{v}_{1j}^2$. Let $\hat{\sigma}_1^2$ be the variance of all $\hat{v}_{1j}^2$ s, and choose α_{1j} and β_{1j} such that the prior distribution Inverse Gamma$(\alpha_{1j}, \beta_{1j})$ has mean $\hat{v}_{1j}^2$ and variance $\hat{\sigma}_1^2$.

We use non-informative priors for row indicator r_i, column indicator e_j and cell-level indicator c_{ij}, i.e., $\pi_{r_i} = \pi_{e_j} = \pi_{c_{ij}} = 1/2$.
