# Supplementary material for: Query Large Scale Microarray Compendium Datasets Using a Model-Based Bayesian Approach with Variable Selection
Source: PLoS One. 2009 Feb 13;4(2):e4495. doi: 10.1371/journal.pone.0004495 (PMC2637418; doi:10.1371/journal.pone.0004495)
Supplement: Material S1 — (0.10 MB PDF) [file pone.0004495.s002.pdf]

Query large scale microarray compendium datasets using a model-based Bayesian approach with variable selection

Ming Hu and Zhaohui S. Qin
Center for Statistical Genetics, Department of Biostatistics, School of Public Health, University of Michigan, Ann Arbor, MI 48109-2029.

Supplementary Material

Table of Content

1. Bayesian Statistical Methods
2. Detailed protocol of microarray data analysis procedure using BEST
3. Query result from six other transcription factors
4. **Table S1.** Performance comparison using area under the curve (AUC) of Receiver Operating Characteristic (ROC) among various methods for querying simulated microarray gene expression datasets.
5. **Table S2.** Information of the 28 potential Lrp target genes identified by BEST when applied to the 100-gene test set selected from the *E. coli* microarray compendium.
6. **Table S3.** Information on the 305 unique experimental conditions (612 different chips with replicates).
7. **Table S4.** Information on the 57 potential Lrp target genes identified by BEST in the 300-gene test set extracted from the *E. coli* compendium.
8. **Table S5.** Information on the 27 potential PdhR target genes identified by BEST in the 100-gene test set extracted from the *E. coli* compendium.
9. **Table S6.** Information on the 31 potential FecI target genes identified by BEST in the 100-gene test set extracted from the *E. coli* compendium.
10. **Table S7.** Information on the 31 potential LexA target genes identified by BEST in the 100-gene test set extracted from the *E. coli* compendium
11. **Table S8.** Information on the 54 potential FlhC target genes identified by BEST in the 200-gene test set extracted from the *E. coli* compendium
12. **Table S9.** Information on the 67 potential FlhD target genes identified by BEST in the 200-gene test set extracted from the *E. coli* compendium
13. **Table S10.** Information on the 56 potential FliA target genes identified by BEST in the 200-gene test set extracted from the *E. coli* compendium
14. **Figure S1.** ROC curves for various query methods when applying to synthetic datasets simulated under different settings and when there are 100% foreground columns
15. **Figure S2.** ROC curves for various query methods when applying to synthetic datasets simulated under different settings and when there are 75% foreground columns.
16. **Figure S3.** ROC curves for various query methods when applying to synthetic datasets simulated under different settings and when there are 50% foreground columns.

17. **Figure S4.** Log-likelihood trace plots of the ten parallel chains resulted from the BEST run on 100-gene and 300-gene test sets selected from the *E. coli* microarray compendium.
18. **Figure S5.** Sequence logo plot [1] and position specific weight matrix (PSWM) for the motif of transcription factor Lrp.
19. **Figure S6.** Boxplots of Pearson correlation coefficients.
20. **Figure S7. A.** Histograms of expression profile differences (z_{ij}) in the top five experimental conditions (foreground). **B.** Histogram of expression profile differences (z_{ij}) in the bottom five experimental conditions (background). Data used here is the 100-gene test set selected from the *E. coli* microarray compendium.
21. **Figure S8.** Trace plots of 24 predicted Lrp target genes identified by BEST that are not in the RegulonDB target set (Part 1). Black lines indicate the query gene—Lrp, the red line indicate the potential target genes. Only the 139 foreground experimental conditions identified by BEST were shown in these plots.
22. **Figure S9.** Trace plots of 24 predicted Lrp target genes identified by BEST that are not in the RegulonDB target set (Part 2). Black lines indicate the query gene—Lrp, the red line indicate the potential target genes. Only the 139 foreground experimental conditions identified by BEST were shown in these plots.
23. **Figure S10.** Trace plots of 24 predicted Lrp target genes identified by BEST that are not in the RegulonDB target set (Part 3). Black lines indicate the query gene—Lrp, the red line indicate the potential target genes. Only the 139 foreground experimental conditions identified by BEST were shown in these plots.
24. **Figure S11.** Trace plots of 24 predicted Lrp target genes identified by BEST that are not in the RegulonDB target set (Part 4). Black lines indicate the query gene—Lrp, the red line indicate the potential target genes. Only the 139 foreground experimental conditions identified by BEST were shown in these plots.
25. **Figure S12.** Trace plots of 24 predicted Lrp target genes identified by BEST that are not in the RegulonDB target set (Part 5). Black lines indicate the query gene—Lrp, the red line indicate the potential target genes. Only the 139 foreground experimental conditions identified by BEST were shown in these plots.
26. **Figure S13.** Trace plots of 24 predicted Lrp target genes identified by BEST that are not in the RegulonDB target set (Part 6). Black lines indicate the query gene—Lrp, the red line indicate the potential target genes. Only the 139 foreground experimental conditions identified by BEST were shown in these plots.
27. **Figure S14. A.** Histograms of expression profile differences (z_{ij}) in the top five experimental conditions (foreground). **B.** Histogram of expression profile differences (z_{ij}) in the bottom five experimental conditions (background). Data used here is the 300-gene test set selected from the *E. coli* microarray compendium.
28. **Additional references.**

1. Bayesian Statistical Methods

The Bayesian Expression Search Tool (BEST) algorithm proposed in the manuscript is a Markov chain Monte Carlo (MCMC)-based computational method [2] which assumed an explicit statistical model that describes the relationship between the query gene and the entire microarray compendium. In this section, we first describe the general Bayesian inference procedure and then the detail implementation for BEST.

The full process of a typical Bayesian analysis can be described as consisting of three main steps [3]. (a) setting up a full probability model, the joint distribution, that captures the relationship among all the variables (e.g., observed data, missing data, unknown parameters) in consideration; (b) summarizing the findings for particular quantities of interest by appropriate posterior distributions, which is typically a conditional distribution of the quantities of interest given the observed data; and (c) evaluating the appropriateness the model and suggesting improvements (model criticism and selection).

A standard procedure for carrying out step (a) is to formulate the scientific question of interest though the use of a probabilistic model, from which we can write down the likelihood function of unknown parameter. In BEST, the input data for statistical inference is the difference, $z = (z_1, z_2, \ldots, z_N)^t$, between the particular query expression profile and the expression profile of genes in the database. The unknown parameters $\Theta = (R, E)$ are a row indicator vector $R = (r_1, r_2, \ldots, r_N)$ and a column indicator vector $E = (e_1, e_2, \ldots, e_M)$. Here R indicates whether the target genes are functionally related to the query gene, and E indicates whether co-expression occurs under the experimental conditions. We assume that the differences between a related gene and the query gene at the foreground columns follow normal distributions, and others follow a background normal distribution. So the query problem could be viewed as statistical inference from a Gaussian mixture model. Then a prior distribution $f_0(\theta)$ is contemplated, which should be both mathematically tractable and scientifically meaningful. In BEST, we adopt standard conjugate priors for these model parameters [3]. The joint probability distribution can then be represented as $Joint = likelihood \times prior$, i.e.,

$$p(y, \theta) = p(y \mid \theta) f_0(\theta)$$

Step (b) is completed by obtaining the posterior distribution through the application of Bayes theorem:

$$p(\theta \mid y) = \frac{p(y, \theta)}{p(y)} = \frac{p(y \mid \theta) f_0(\theta)}{\int p(y \mid \theta) f_0(\theta) d\theta} \propto p(y \mid \theta) f_0(\theta)$$

After integrating out nuisance parameters, we get full conditional distributions of R and E, which are Bernoulli distributions. Finding the maximum likelihood estimator seems a good way to solve this problem, i.e., finding the genes which are most likely functionally related to the query gene, and the experimental conditions where co-expression most likely occurs. However, it is impossible to enumerate all possible combinations due to the large numbers of genes in database and so many different experimental conditions. We therefore employ a MCMC strategy to find a near-optimal solution. MCMC refers a collection of stochastic simulation techniques that can be used to sample from complicated probability distributions. Its basic principle is to design a Markov transition rule so that the equilibrium distribution of the

Markov chain is the desired distribution. One of the most popular algorithms is the Gibbs sampler, which updates one component at a time. Each component-wise update is achieved by a sample drawn from its conditional distribution with values of other components held fixed. In BEST, we use Gibbs sampler to sample joint distribution of row indicator vector $R = (r_1, r_2, \ldots, r_N)$ and column indicator vector $E = (e_1, e_2, \ldots, e_M)$. We draw Bernoulli sample of each indicator according to the Bayes factor between two normal distributions, with all the other indicators held constant. The posterior likelihood converges after a number of iterations, and then we report the parameters corresponding to the posterior mode as our parameter estimators. Several parallel chains are used simultaneously to prevent trapping in the local mode.

The Bayesian approach has at least two advantages. First, through the prior distribution we can use prior knowledge and information about the value of unknown parameters. This is especially important since biologists often have substantial knowledge about the subject under study. To the extent that this information is correct, it will sharpen the inference about the unknown parameters. In BEST, users can customize the parameters for prior distributions such that certain genes and experimental conditions will have a higher chance to be selected as target gene/foreground conditions. Users can even fix some indicator variables based on previous knowledge/experience. Correctly assigned informative priors can make BEST converge much faster and generate much more accurate predictions.

Second, treating all the variables in the system as random variables greatly clarifies the methods of analysis. It follows from the basic probability theory (Bayes formula) that information about the realized value of any random variable based on observation of related random variables is summarized in the conditional distribution. In BEST, we can get the close form of posterior distribution since we adopt standard conjugate priors. In addition, it is easy to integrate out nuisance parameters and results in simple distribution (Bernoulli distribution) for parameters of interest.

2. Detailed protocol of microarray data analysis procedure using BEST

The *E. coli* dataset originally came from the study reported in Faith et al. (2007). The authors conducted a comprehensive survey of gene expression profiles of all *E. coli* genes using 612 Affymetrix GeneChip arrays treated with 305 different experimental conditions. RMA normalized data (Faith et al., 2008) was used in this study, which consisted of 4,217 genes and 305 samples. The detail of microarray data analysis procedure, such as microarray profiling, bacterial strains, steady-state experiments, time-course experiments, preparation of RNA and hybridization, external data, microarray normalization, are available at (Faith et al., 2007).

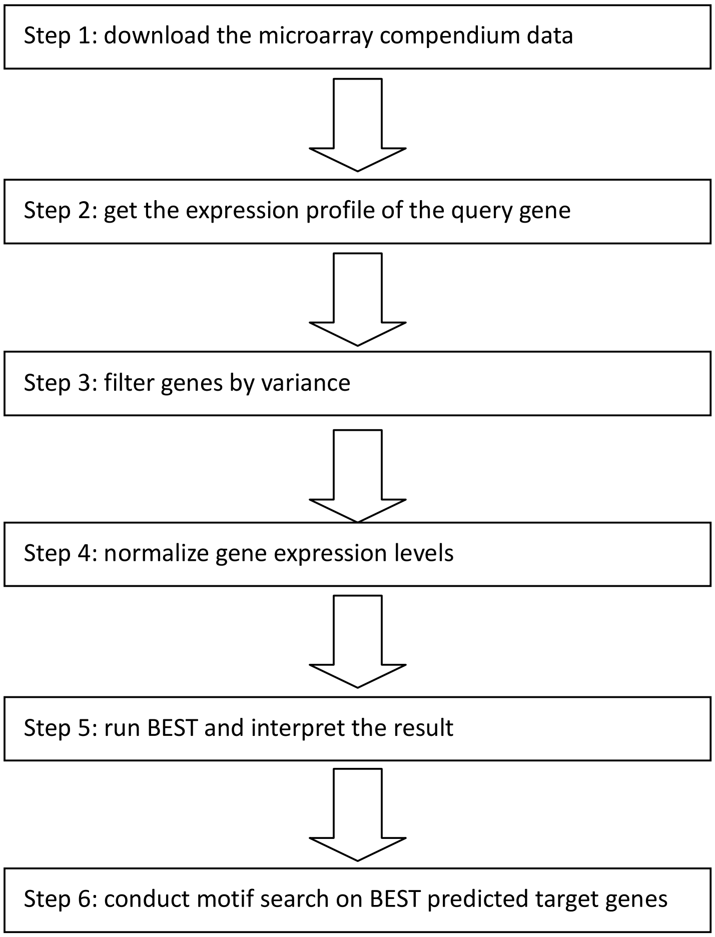

Step 1: download microarray compendium data file “E_coli_v4_Builid_4_norm.tar.gz” from http://m3d.bu.edu/norm/?C=M;O=A. This zipped data file describes the normalized compendium dumps from M3D, which contains six files with expression data. “avg_E_coli_v4_Build_4_exps305probes4217.tab”, the expression data file which contains 305 experimental conditions and 4217 genes, was used in our study.

Step 2: get the expression profile of the query gene, for example: Lrp, from the microarray compendium “avg_E_coli_v4_Build_4_exps305probes4217.tab”, which is the expression profile of Lrp across the 305 different experimental conditions.

Step 3: filter genes based on their variances. First, we calculated the variances of all 4217 genes found in the microarray compendium. We then remove all genes whose variation across all experimental conditions is less than the query gene. This purpose of filtering is to reduce computation time and to maximize the chance of finding biological meaningful targets. For the query gene Lrp, there are 524 genes (out of 4217 in total, 12%) with total expression variance greater than that of Lrp. We thus used these 524 candidate genes in our search.

Step 4: normalize the query gene and the 524 candidate genes. First, we calculated the mean and standard deviation across the 305 experimental conditions for each gene, and then normalize each of the gene expression levels by subtracting its mean and dividing by its standard deviation. After normalization, the query gene Lrp and the 524 candidate genes all have the same mean and variance (mean=0 and standard deviation=1).

Step 5: run BEST on the normalized gene expression levels using user-specified parameters such as the number of iteration in MCMC and the number of parallel chains.

Step 6: conduct motif search. We download position specific weight matrices (PSWM) from RegulonDB (http://regulondb.ccg.unam.mx/data/Matrix_AlignmentSet.txt), and the complete *E. coli* genome from GenBank (ftp://ftp.ncbi.nih.gov/genbank/genomes/Bacteria/Escherichia_coli_K12_substr__MG1655/U00096.fna). We then calculated the log likelihood ratio comparing between the motif model and the background model on each possible start location in the intergenic regions (up to 500 bp upstream) of genes identified by BEST. The locations with log likelihood ratio higher than a certain threshold are treated as putative motifs.

3. Query result from six other transcription factors

In addition to TF Leucine-responsive Regulatory Protein (Lrp), we used another six TFs (PdhR, FecI, LexA, FlhC, FlhD and FliA) as the query genes in this study to test BEST's performance. All six TFs have an almost equal number of target genes in ReglonDB and CLR prediction with an estimated 60% precision (Faith et al. 2007).

3.1. Query result from PdhR

PdhR has five target genes in ReglonDB. CLR predicted four target genes with 60% precision. None of them is in the RegulonDB target set. We included these nine genes and another 91 negative genes to form a 100 gene test set. BEST found 27 target genes and 179 experimental conditions as foreground. Twenty-seven of BEST's target genes included two target genes in ReglonDB and four target genes predicted by CLR (p-value of 0.0110). We found three genes (uspE, cspD, aceA) with inversed pattern. Table S4 lists all PdhR target genes identified by BEST.

3.2. Query result from FecI

FecI has six target genes in ReglonDB. CLR predicted eight target genes with 60% precision. Eight of these nine predictions are not in the RegulonDB target set. We included these 13 genes and another 87 negative genes to form a 100 gene test set. BEST found 31 target genes and 169 experimental conditions as foreground. Thirty-one of BEST's target genes included all 13 target genes in ReglonDB and target genes predicted by CLR (p-value of 2.9×10^{-8}). We found no gene with inversed pattern. Table S5 lists all FecI target genes identified by BEST.

3.3. Query result from LexA

LexA has 16 target genes. CLR predicted 17 targets genes with 60% precision. 10 of these 17 predictions are not in the RegulonDB target set. We included these 26 genes and another 74 negative genes to form a 100 gene test set. BEST found 31 target genes and 237 experimental conditions as foreground. Thirty-one of BEST's target genes included 10 target genes in ReglonDB and all target genes predicted by CLR (p-value of 1.5×10^{-8}). We found one gene (uspE) with inversed pattern. Table S6 lists all LexA target genes identified by BEST.

3.4. Query result from FlhC

FlhC has 30 target genes in ReglonDB. CLR predicted 53 targets genes with 60% precision. 24 of these 53 predictions are not in the RegulonDB target set. We included these 54 genes and another 146 negative genes to form a 200 gene test set. BEST found 54 target genes and 266 experimental conditions as foreground. Fifty-four of BEST's target genes included 29 target genes in ReglonDB and all target predicted by CLR (p-value of 2.7×10^{-46}). We found no gene with inversed pattern. yjdA is the new hypothetical FlhC target gene identified by BEST in addition to false positive genes in Faith's prediction with 60% precision. Table S7 lists all FlhC target genes identified by BEST.

3.5. Query result from FlhD

FlhD has 46 target genes in ReglonDB. CLR predicted 46 target genes with 60% precision. Twenty of these 46 predictions are not in the RegulonDB target set. We included these 66 genes

and another 134 negative genes to form a 200 gene test set. BEST found 55 target genes and 215 experimental conditions as foreground. Fifty-five of BEST's target genes included 29 target genes in ReglonDB and all target genes predicted by CLR (p-value of 1.67×10^{-17}). We found two genes (micF, gadX) with inversed pattern. cheY, cheZ, flxA, micF, gadX and yjdA are the six new hypothetical FlhD target genes identified by BEST in addition to false positive genes predicted by CLR with 60% precision. Table S8 lists all FlhC target genes identified by BEST.

3.6. Query result from FliA

FliA has 42 target genes in ReglonDB. CLR predicted 56 target genes with 60% precision. Fifteen of these 56 predictions are not in the RegulonDB target set. We included these 57 genes and another 143 negative genes to form a 200 gene test set. BEST found 56 target genes and 281 experimental conditions as foreground. Fifty-six of BEST's target genes included 41 genes in ReglonDB and all target predicted by CLR (p-value of 4.08×10^{-47}). We found no genes with inversed pattern, and no new hypothetical FliA target gene identified by BEST in addition to false positive genes predicted by CLR with 60% precision. Table S9 list all FliA target genes identified by BEST.

Additional references:

1. Schneider TD, Stephens RM (1990) Sequence logos: a new way to display consensus sequences. Nucleic Acids Res 18: 6097-6100.
2. Liu JS (2001) Monte Carlo Strategies in Scientific Computing. Berlin: springer Verlag.
3. Gelman A, Carlin JB, Stern HS, Rubin DB (1995) Bayesian data analysis. London: Chapman & Hall. xix, 526 p.
4. Bembom O (2007) Sequence logos for DNA sequence alignments.
