# Supplementary material for: Query Large Scale Microarray Compendium Datasets Using a Model-Based Bayesian Approach with Variable Selection
Source: PLoS One. 2009 Feb 13;4(2):e4495. doi: 10.1371/journal.pone.0004495 (PMC2637418; doi:10.1371/journal.pone.0004495)
Supplement: Table S1 — (0.02 MB DOC) [file pone.0004495.s003.doc]

**Table S1.** Performance comparison using area under the curve (AUC) of Receiver Operating Characteristic (ROC) among various methods for querying simulated microarray gene expression datasets. Best results are displayed in bold.

|  |  |  |  |  |  |  |  |  |  |
| --- | --- | --- | --- | --- | --- | --- | --- | --- | --- |
| Case | Sub-case^*^ | Pearson^a^ | Spearman^b^ | Kendall^c^ | QDB^d^ | Mutual^e^ | BEST A^f^ | BEST B^g^ | BEST C^h^ |
| Case 1: | I | **1** | **1** | **1** | **1** | **1** | **1** | **1** | **1** |
| 100% | II | 0.68 | 0.68 | 0.68 | 0.85 | **1** | **1** | **1** | **1** |
| foreground | III | **1** | **1** | **1** | **1** | **1** | **1** | **1** | **1** |
|  | IV | 0.66 | 0.71 | 0.71 | 0.79 | 0.85 | **1** | **1** | **1** |
| Case 2: | I | 0.98 | **1** | **1** | **1** | 0.98 | **1** | **1** | **1** |
| 75% | II | 0.7 | 0.71 | 0.71 | 0.89 | 0.95 | **1** | **1** | **1** |
| foreground | III | 0.99 | **1** | **1** | **1** | 0.98 | **1** | **1** | **1** |
|  | IV | 0.67 | 0.71 | 0.71 | 0.84 | 0.88 | **1** | **1** | **1** |
| Case 3: | I | 0.89 | 0.93 | 0.95 | 0.99 | 0.87 | **1** | **1** | **1** |
| 50% | II | 0.67 | 0.69 | 0.69 | 0.89 | 0.81 | **1** | **1** | **1** |
| foreground | III | 0.88 | 0.92 | 0.94 | 0.92 | 0.84 | **1** | **1** | **1** |
|  | IV | 0.59 | 0.62 | 0.64 | 0.77 | 0.69 | 0.98 | **0.99** | **0.99** |
| Case 4: | I | 0.68 | 0.69 | 0.71 | 0.55 | 0.61 | 0.91 | 0.98 | **0.99** |
| 25% | II | 0.55 | 0.55 | 0.56 | 0.51 | 0.58 | 0.85 | 0.92 | **0.99** |
| foreground | III | 0.67 | 0.68 | 0.69 | 0.51 | 0.59 | 0.93 | 0.98 | **0.99** |
|  | IV | 0.54 | 0.55 | 0.56 | 0.53 | 0.51 | 0.79 | 0.84 | **0.88** |
|  |  |  |  |  |  |  |  |  |  |

^*^ There are four sub-cases in each of the simulated cases with the same amount of foreground columns.

Sub case I: no linear transformation;

Sub case II: only add linear transformation;

Sub case III: only add cell-level noise;

Sub case IV: add both linear transformation and cell-level noise.

^a^ Query method using Pearson correlation coefficient.

^b^ Query method using Spearman correlation coefficient.

^c^ Query method using Kendall’s *τ*.

^d^ Query method using QDB.

^e^ Query method using mutual information.

^f^ Query method using BEST.

^g^Query method using BEST allowing exclusion of individual cells from the foreground.

^h^ Query method using BEST when fixing the indicator variables of five true target genes and five true experimental conditions as 1.
