# Supplementary material for: Query Large Scale Microarray Compendium Datasets Using a Model-Based Bayesian Approach with Variable Selection
Source: PLoS One. 2009 Feb 13;4(2):e4495. doi: 10.1371/journal.pone.0004495 (PMC2637418; doi:10.1371/journal.pone.0004495)
Supplement: Table S2 — (0.02 MB DOC) [file pone.0004495.s004.doc]

**Table S2.** Information of the 28 potential Lrp target genes identified by BEST when applied to the 100-gene test set selected from the *E. coli* microarray compendium.

|  |  |  |  |  |  |
| --- | --- | --- | --- | --- | --- |
| Rank | Gene Name ^a^ | Log Bayes Ratio | positive/negative ^b^ | RegulonDB ^c^ | CLR ^d^ |
| 1 | serA | 131.81 |  | X | X |
| 2 | leuA | 129.99 |  | X | X |
| 3 | leuL | 128.72 |  | X | X |
| 4 | gltD | 128.22 |  | X | X |
| 5 | leuD | 123.19 |  | X | X |
| 6 | ilvI | 120.44 |  | X |  |
| 7 | ilvH | 119.61 |  | X | X |
| 8 | gltB | 119.04 |  | X |  |
| 9 | leuC | 116.22 |  | X | X |
| 10 | livG | 115.37 |  | X | X |
| 11 | ilvE | 114.30 |  | X |  |
| 12 | livK | 113.30 |  | X | X |
| 13 | leuB | 110.28 |  | X | X |
| 14 | livJ | 109.72 |  | X |  |
| 15 | livM | 108.48 |  | X |  |
| 16 | gcvB | 107.80 | negative |  |  |
| 17 | serC | 103.20 |  | X | X |
| 18 | aroA | 97.58 |  | X | X |
| 19 | livH | 94.76 |  | X | X |
| 20 | livF | 93.82 |  | X | X |
| 21 | ilvL | 90.43 |  | X |  |
| 22 | ilvD | 89.88 |  | X |  |
| 23 | lysU | 84.52 | negative | X |  |
| 24 | kbl | 81.47 | negative | X |  |
| 25 | tdh | 80.09 | negative | X |  |
| 26 | ilvG | 79.47 |  | X |  |
| 27 | ilvM | 71.23 |  | X | X |
| 28 | ilvA | 65.02 |  | X |  |
|  |  |  |  |  |  |

^a^ Genes displayed here are sorted by the Log Bayes ratio (target gene versus non-target gene).

^b^ Blank indicates that the target gene shows the same pattern as the query gene. Negative indicates that the target gene shows the inversed pattern as the query gene.

^c^ BEST indentifies 27 genes among 61 target genes in RegulonDB. “X” indicates that the predicted gene is in the RegulonDB target set.

^d^ “X” indicates that the gene is predicted by CLR as a target gene.
