# Supplementary material for: Query Large Scale Microarray Compendium Datasets Using a Model-Based Bayesian Approach with Variable Selection
Source: PLoS One. 2009 Feb 13;4(2):e4495. doi: 10.1371/journal.pone.0004495 (PMC2637418; doi:10.1371/journal.pone.0004495)
Supplement: Table S3 — (0.04 MB DOC) [file pone.0004495.s005.doc]

**Table S3.** Information on the 305 distinct experimental conditions (among 612 different chips with replicates). The 305 experimental conditions are sorted by log Bayes ratio. BEST predicts the top 143 as foreground and the rest as background. Detail information of these 612 chips and 305 experimental conditions could be found in microarray compendium data file “E_coli_v4_Builid_4_norm.tar.gz” from the Gardner Lab. (<http://m3d.bu.edu/norm/?C=M;O=A>)

|  |  |  |  |
| --- | --- | --- | --- |
| Rank | Experimental Conditions | Replicates | Log Bayes Ratio |
| 1 | M9_K_arcA_anaerobic | 3 | 50.47 |
| 2 | M9_WT | 3 | 48.79 |
| 3 | M9_K_appY_anaerobic | 3 | 44.81 |
| 4 | M9_K_soxS_anaerobic | 3 | 43.21 |
| 5 | M9_K_arcA | 3 | 38.07 |
| 6 | M9_K_oxyR_anaerobic | 3 | 37.60 |
| 7 | M9_K_soxS | 3 | 37.23 |
| 8 | M9_K_arcAfnr | 3 | 37.02 |
| 9 | M9_WT_anaerobic | 4 | 35.75 |
| 10 | M9_K_oxyR | 3 | 35.52 |
| 11 | lacZ_W1863_t0 | 1 | 35.24 |
| 12 | M9_K_fnr | 3 | 34.34 |
| 13 | ccdB_MG1655_t30 | 2 | 34.14 |
| 14 | M9_K_fnr_anaerobic | 3 | 34.05 |
| 15 | ccdB_W1872_t60 | 1 | 31.42 |
| 16 | lacZ_W1863_t60 | 1 | 31.11 |
| 17 | ccdB_W1872_t30 | 1 | 30.66 |
| 18 | ccdB_W1872_t90 | 1 | 29.37 |
| 19 | M9_K_appY | 3 | 29.01 |
| 20 | lacZ_MG1063_t0 | 2 | 27.61 |
| 21 | ccdB_MG1655_t0 | 2 | 26.92 |
| 22 | norfloxacin_chelator_MG1063_t0 | 1 | 26.75 |
| 23 | ccdB_chelator_MG1063_t0 | 1 | 26.31 |
| 24 | ccdB_chelator_MG1063_t60 | 1 | 26.12 |
| 25 | lacZ_W1863_t30 | 1 | 25.47 |
| 26 | fnr_K_fnrAnaerobic | 4 | 25.21 |
| 27 | cybr_N_log | 2 | 24.95 |
| 28 | MG1063_uninduced_t0 | 1 | 24.50 |
| 29 | MG1063_uninduced_t60 | 1 | 24.36 |
| 30 | MG1063_uninduced_t30 | 1 | 24.22 |
| 31 | suspension_7hr | 1 | 23.49 |
| 32 | MG1655_ampicillin_t120 | 1 | 23.40 |
| 33 | ccdB_chelator_W1872_t0 | 1 | 23.30 |
| 34 | MG1655_ampicillin_t30 | 1 | 23.23 |
| 35 | ik_H2_T3 | 1 | 23.04 |
| 36 | fnr_wtAnaerobic | 3 | 22.85 |
| 37 | ik_L2_T3.5 | 1 | 22.80 |
| 38 | norfloxacin_BW25113_t120 | 1 | 22.59 |
| 39 | norfloxacin_BW25113recA_t60 | 1 | 22.39 |
| 40 | har_S1_R_noIPTG | 3 | 21.62 |
| 41 | ccdB_chelator_MG1063_t30 | 1 | 21.60 |
| 42 | cybr_O_log | 2 | 21.56 |
| 43 | biofilm_4hr | 1 | 21.32 |
| 44 | ph5.7_anaerobic | 5 | 21.24 |
| 45 | carbonSourceForaging | 2 | 21.02 |
| 46 | WT_MOPS_glucose | 5 | 20.77 |
| 47 | ik_L2_T4 | 1 | 20.38 |
| 48 | M9_K_arcAfnr_anaerobic | 3 | 20.28 |
| 49 | WT_MOPS_glycerol | 2 | 20.08 |
| 50 | ik_H2_T3.5 | 1 | 19.86 |
| 51 | MG1655_uninduced_t0 | 1 | 19.36 |
| 52 | luc2_U_N0000 | 2 | 19.30 |
| 53 | MG1655_ampicillin_t60 | 1 | 18.85 |
| 54 | ccdB_BW25113recA_t120 | 1 | 18.84 |
| 55 | ik_L2_T3 | 1 | 18.77 |
| 56 | menC_U_N0075 | 3 | 18.76 |
| 57 | crcB_U_N0075 | 3 | 18.69 |
| 58 | MG1655_t480_aerobic | 2 | 18.61 |
| 59 | cpxR_U_N0075 | 3 | 18.31 |
| 60 | era_U_N0075 | 3 | 18.26 |
| 61 | crp_U_N0075 | 3 | 18.09 |
| 62 | luc_U_N0075 | 3 | 18.00 |
| 63 | gcvR_U_N0075 | 3 | 17.83 |
| 64 | dnaA_U_N0075 | 3 | 17.79 |
| 65 | menB_U_N0075 | 3 | 17.65 |
| 66 | fis_U_N0075 | 3 | 17.62 |
| 67 | ccdB_BW25113recA_t30 | 1 | 17.60 |
| 68 | ccdB_MG1063_t0 | 2 | 17.18 |
| 69 | ik_H2_T2.5 | 1 | 17.04 |
| 70 | WT_N0075 | 2 | 17.04 |
| 71 | ccdB_W1872_t0 | 1 | 16.89 |
| 72 | MG1655_t720_aerobic | 2 | 16.86 |
| 73 | norfloxacin_BW25113recA_t30 | 1 | 16.59 |
| 74 | MG1655_t270_anaerobic | 2 | 16.35 |
| 75 | lacZ_W1863_t90 | 1 | 16.27 |
| 76 | rimI_U_N0075 | 3 | 16.19 |
| 77 | minD_U_N0075 | 3 | 16.15 |
| 78 | zipA_U_N0075 | 3 | 15.99 |
| 79 | dinP_U_N0025 | 3 | 15.75 |
| 80 | lacZ_MG1063_t30 | 2 | 15.74 |
| 81 | mcrB_U_N0075 | 3 | 15.62 |
| 82 | ccdB_BW25113recA_t180 | 1 | 15.53 |
| 83 | MG1655_uninduced_t60 | 1 | 15.48 |
| 84 | norfloxacin_chelator_MG1063_t0.1 | 1 | 15.34 |
| 85 | yebF_U_N0075 | 3 | 15.21 |
| 86 | MG1655_t150_aerobic | 2 | 15.13 |
| 87 | ccdB_BW25113_t180 | 1 | 15.01 |
| 88 | biofilm_wt_noGlucose | 1 | 14.83 |
| 89 | MG1655_t225_anaerobic | 2 | 14.75 |
| 90 | ccdB_BW25113_t30 | 1 | 14.61 |
| 91 | ast_pBADsup2 | 3 | 14.50 |
| 92 | ph7 | 5 | 14.17 |
| 93 | recA_U_N0025 | 3 | 13.88 |
| 94 | MG1655_t300_aerobic | 2 | 13.71 |
| 95 | uspA_U_N0075 | 3 | 13.53 |
| 96 | norfloxacin_MG1063_t0 | 1 | 13.47 |
| 97 | mcrC_U_N0075 | 3 | 13.41 |
| 98 | bcp_U_N0075 | 3 | 13.22 |
| 99 | b2618_U_N0075 | 3 | 13.19 |
| 100 | pepAA_t0 | 2 | 13.00 |
| 101 | nupC_U_N0075 | 3 | 12.84 |
| 102 | ldrA_U_N0075 | 3 | 12.34 |
| 103 | ccdB_K12_t90 | 1 | 12.15 |
| 104 | lacZ_K12_t60 | 1 | 12.09 |
| 105 | BW25113_uninduced_t120 | 1 | 11.80 |
| 106 | yoeB_U_N0075 | 3 | 11.71 |
| 107 | minE_U_N0075 | 3 | 11.48 |
| 108 | ph7_anaerobic | 5 | 11.45 |
| 109 | luc_U_N0025 | 3 | 11.44 |
| 110 | ast_pBAD18 | 3 | 11.42 |
| 111 | MG1655_t405_aerobic | 2 | 11.28 |
| 112 | sbcB_U_N0075 | 3 | 10.53 |
| 113 | fklB_U_N0075 | 3 | 10.45 |
| 114 | lacZ_MG1655_t0 | 1 | 10.36 |
| 115 | MG1655_t1080_aerobic | 2 | 9.96 |
| 116 | gyrA_U_N0075 | 3 | 9.66 |
| 117 | har_S0_R_noIPTG | 5 | 9.52 |
| 118 | norfloxacin_BW25113_t180 | 1 | 9.51 |
| 119 | suspension_4hr | 1 | 9.45 |
| 120 | W3110_K_luxS | 2 | 9.23 |
| 121 | murI_U_N0075 | 3 | 8.80 |
| 122 | MG1655_uninduced_t120 | 1 | 8.43 |
| 123 | BW25113recA_uninduced_t180 | 1 | 7.97 |
| 124 | folA_U_N0075 | 3 | 7.76 |
| 125 | lacZ_K12_t30 | 1 | 7.69 |
| 126 | cspF_U_N0075 | 3 | 7.61 |
| 127 | dnaN_U_N0075 | 3 | 6.63 |
| 128 | ccdB_BW25113recA_t0 | 1 | 6.57 |
| 129 | W3110_wt | 2 | 6.43 |
| 130 | ccdB_K12_t30 | 1 | 5.91 |
| 131 | hlpA_U_N0075 | 3 | 5.48 |
| 132 | MG1655_uninduced_t30 | 1 | 4.20 |
| 133 | biofilm_7hr | 1 | 3.80 |
| 134 | T60_N10000 | 3 | 3.16 |
| 135 | MG1655_t180_anaerobic | 2 | 2.78 |
| 136 | WT_N0000 | 2 | 2.69 |
| 137 | dinI_U_N0025 | 3 | 0.68 |
| 138 | BW25113recA_uninduced_t120 | 1 | 0.29 |
| 139 | ccdB_BW25113recA_t60 | 1 | 0.29 |
| 140 | ik_L2_T4.5 | 1 | 0.22 |
| 141 | dam_U_N0075 | 3 | 0.05 |
| 142 | lacZ_MG1655_t60 | 1 | -0.22 |
| 143 | rstB_U_N0075 | 3 | -0.71 |
| 144 | pepAA_t30 | 2 | -1.03 |
| 145 | dnaT_U_N0075 | 3 | -1.16 |
| 146 | MGD1_t30 | 2 | -1.26 |
| 147 | norfloxacin_BW25113recA_t0 | 1 | -1.62 |
| 148 | gyrI_U_N0075 | 3 | -1.83 |
| 149 | MG1655_kanamycin_t120 | 1 | -1.95 |
| 150 | ik_H2_T8 | 1 | -2.05 |
| 151 | sulA_U_N0025 | 3 | -2.29 |
| 152 | umuD_U_N0025 | 3 | -2.38 |
| 153 | ccdB_K12_t60 | 1 | -2.44 |
| 154 | T0_N0000 | 3 | -2.50 |
| 155 | ik_H2_T4.5 | 1 | -2.56 |
| 156 | norfloxacin_BW25113_t30 | 1 | -2.93 |
| 157 | lacZ_MG1655_t90 | 1 | -2.93 |
| 158 | ik_H2_T6 | 1 | -3.02 |
| 159 | ik_L2_T2.5 | 1 | -3.04 |
| 160 | ruvA_U_N0025 | 3 | -3.06 |
| 161 | ccdB_BW25113_t0 | 1 | -3.09 |
| 162 | lexA_U_N0025 | 3 | -3.54 |
| 163 | ik_L2_T5.5 | 1 | -3.60 |
| 164 | holD_U_N0075 | 3 | -3.87 |
| 165 | nrdA_U_N0075 | 3 | -4.06 |
| 166 | ccdB_K12_t120 | 1 | -4.15 |
| 167 | WT_D_N0100 | 2 | -4.29 |
| 168 | pepCO_t30 | 2 | -4.37 |
| 169 | lon_U_N0025 | 3 | -4.56 |
| 170 | recA_D_N0100 | 2 | -4.60 |
| 171 | MG1655_kanamycin_t60 | 1 | -4.62 |
| 172 | MG1655_kanamycin_t30 | 1 | -4.65 |
| 173 | ccdB_BW25113_t60 | 1 | -4.90 |
| 174 | galF_U_N0075 | 3 | -5.04 |
| 175 | ruvC_U_N0075 | 3 | -5.24 |
| 176 | T48_N10000 | 3 | -5.43 |
| 177 | MG1063_uninduced_t120 | 1 | -5.44 |
| 178 | ccdB_chelator_W1872_t30 | 1 | -5.66 |
| 179 | lacZ_MG1063_t90 | 2 | -5.84 |
| 180 | BW25113recA_uninduced_t0 | 1 | -5.95 |
| 181 | uvrA_U_N0025 | 3 | -6.13 |
| 182 | IHF_U_N0075 | 2 | -6.15 |
| 183 | MOPS_K_dps_stationary2 | 1 | -6.15 |
| 184 | relA_U_N0025 | 3 | -6.27 |
| 185 | BW25113recA_uninduced_t30 | 1 | -7.01 |
| 186 | emrR_U_N0075 | 2 | -7.21 |
| 187 | ik_H2_T4 | 1 | -7.22 |
| 188 | cybr_O | 2 | -7.24 |
| 189 | WT_MOPS_stationary3 | 2 | -7.39 |
| 190 | WT_MOPS_heatShock | 1 | -7.60 |
| 191 | MOPS_K_dps_stationary | 2 | -7.61 |
| 192 | cybr_N | 2 | -7.66 |
| 193 | luc_U_N0000 | 3 | -7.72 |
| 194 | ph8.5_anaerobic | 5 | -7.86 |
| 195 | pyrC_U_N0075 | 3 | -8.00 |
| 196 | ph5 | 5 | -8.10 |
| 197 | BW25113_uninduced_t30 | 1 | -8.33 |
| 198 | cybr_N_stat | 2 | -8.51 |
| 199 | WT_MOPS_stationary2 | 2 | -8.53 |
| 200 | hscA_U_N0075 | 3 | -8.60 |
| 201 | ik_L2_T6 | 1 | -8.67 |
| 202 | ik_H2_T5.5 | 1 | -8.94 |
| 203 | mazF_U_N0025 | 3 | -9.11 |
| 204 | pET3d_t0 | 2 | -9.19 |
| 205 | luc2_U_N0025 | 2 | -9.21 |
| 206 | T24_N10000 | 3 | -9.22 |
| 207 | nrdB_U_N0075 | 2 | -9.36 |
| 208 | BW25113_uninduced_t180 | 1 | -9.42 |
| 209 | T36_N10000 | 3 | -9.74 |
| 210 | MG1655_spectinomycin_t30 | 1 | -9.74 |
| 211 | norfloxacin_BW25113_t60 | 1 | -9.75 |
| 212 | WT_MOPS_cipro2 | 1 | -10.61 |
| 213 | ccdB_MG1655_t60 | 2 | -10.66 |
| 214 | MGD1_t0 | 2 | -10.98 |
| 215 | WT_MOPS_acetate | 2 | -10.99 |
| 216 | ccdB_chelator_MG1063_t120 | 1 | -11.15 |
| 217 | ccdB_MG1063_t30 | 2 | -11.39 |
| 218 | lacZ_K12_t120 | 1 | -11.41 |
| 219 | cybr_O_stat | 2 | -11.44 |
| 220 | W3110_K_luxS_glucose | 1 | -11.44 |
| 221 | lacZ_MG1063_t60 | 2 | -11.71 |
| 222 | ccdB_BW25113_t120 | 1 | -11.87 |
| 223 | ccdB_MG1655_t90 | 2 | -11.91 |
| 224 | ik_L2_T5 | 1 | -12.14 |
| 225 | WT_MOPS_proline | 2 | -12.22 |
| 226 | recA_D_N0050 | 2 | -12.34 |
| 227 | WT_MOPS_stationary4 | 2 | -12.42 |
| 228 | WT_MOPS_cipro | 1 | -12.81 |
| 229 | WT_N0025 | 2 | -12.92 |
| 230 | T12_N10000 | 3 | -13.07 |
| 231 | lacZ_MG1063_120 | 1 | -13.07 |
| 232 | BW25113recA_uninduced_t60 | 1 | -13.11 |
| 233 | fnr_K_fnrAerobic | 3 | -13.21 |
| 234 | MG1655_t150_anaerobic | 2 | -13.42 |
| 235 | norfloxacin_BW25113_t0 | 1 | -13.55 |
| 236 | recA_D_N0000 | 2 | -13.59 |
| 237 | WT_MOPS_acidShock | 2 | -13.77 |
| 238 | MG1655_spectinomycin_t60 | 1 | -13.82 |
| 239 | MG1655_spectinomycin_t120 | 1 | -14.22 |
| 240 | biofilm_15hr | 1 | -14.39 |
| 241 | K12_t360 | 3 | -14.44 |
| 242 | ccdB_K12_t0 | 1 | -14.44 |
| 243 | WT_MOPS_lateLog | 3 | -14.46 |
| 244 | suspension_15hr | 1 | -15.01 |
| 245 | W3110_wt_glucose | 2 | -15.23 |
| 246 | har_S4_noIPTG | 3 | -15.91 |
| 247 | norfloxacin_BW25113recA_t120 | 1 | -16.73 |
| 248 | ik_H2_T5 | 1 | -16.96 |
| 249 | har_S1_noIPTG | 3 | -16.97 |
| 250 | norfloxacin_MG1063_t30 | 1 | -17.00 |
| 251 | lacZ_MG1655_t30 | 1 | -17.38 |
| 252 | pepCO_t0 | 2 | -17.62 |
| 253 | lacZ_K12_t0 | 1 | -17.85 |
| 254 | lacZ_K12_t90 | 1 | -18.07 |
| 255 | har_S1_IPTG | 3 | -18.16 |
| 256 | MOPS_K_cspA | 1 | -18.20 |
| 257 | MG1655_t86400_cecum | 5 | -18.47 |
| 258 | BW25113_uninduced_t60 | 1 | -18.67 |
| 259 | norfloxacin_chelator_MG1063_t0.2 | 1 | -18.95 |
| 260 | norfloxacin_chelator_MG1063_t0.3 | 1 | -19.00 |
| 261 | MG1655_t1560_aerobic | 2 | -19.27 |
| 262 | biofilm_24hr | 1 | -19.31 |
| 263 | biofilm_K_yceP | 1 | -19.35 |
| 264 | MG1655_norfloxacin_t30 | 1 | -19.60 |
| 265 | MG1655_norfloxacin_t120 | 1 | -19.95 |
| 266 | har_S0_noIPTG | 3 | -20.33 |
| 267 | MOPS_K_dps | 3 | -20.40 |
| 268 | cybr_KNO_N | 2 | -20.71 |
| 269 | har_S4_IPTG | 3 | -21.49 |
| 270 | ph8.7 | 5 | -21.97 |
| 271 | K12_t150_K_fis | 3 | -22.24 |
| 272 | ik_L2_T8 | 1 | -22.32 |
| 273 | T24_N0000 | 3 | -22.43 |
| 274 | pET3d_t30 | 2 | -22.91 |
| 275 | K12_t90_K_fis | 3 | -23.30 |
| 276 | BW25113_uninduced_t0 | 1 | -23.45 |
| 277 | K12_t360_K_fis | 3 | -23.71 |
| 278 | biofilm_K_yceP_indole | 2 | -25.13 |
| 279 | WT_N0050 | 2 | -25.65 |
| 280 | T60_N0000 | 3 | -25.75 |
| 281 | MOPS_K_hupB | 1 | -26.19 |
| 282 | norfloxacin_MG1063_t60 | 1 | -26.30 |
| 283 | WT_MOPS_stationary | 2 | -26.59 |
| 284 | K12_t240_K_fis | 3 | -26.70 |
| 285 | K12_t150 | 3 | -27.19 |
| 286 | har_S4_R_IPTG | 3 | -27.85 |
| 287 | norfloxacin_BW25113recA_t180 | 1 | -29.03 |
| 288 | ccdB_MG1063_t60 | 2 | -29.70 |
| 289 | MG1655_norfloxacin_t60 | 1 | -30.08 |
| 290 | ccdB_MG1063_t120 | 1 | -31.01 |
| 291 | ccdB_chelator_W1872_t60 | 1 | -31.04 |
| 292 | K12_t240 | 3 | -32.33 |
| 293 | MOPS_K_crp | 3 | -32.52 |
| 294 | MG1063_uninduced_t180 | 1 | -32.69 |
| 295 | biofilm_K_tnaA | 1 | -32.85 |
| 296 | MOPS_K_hns | 3 | -33.23 |
| 297 | ccdB_chelator_W1872_t120 | 1 | -33.23 |
| 298 | suspension_24hr | 1 | -33.70 |
| 299 | biofilm_wt_glucose | 1 | -34.55 |
| 300 | har_S1_R_IPTG | 3 | -35.58 |
| 301 | biofilm_K_trpE | 1 | -37.15 |
| 302 | ccdB_MG1063_t90 | 2 | -37.89 |
| 303 | norfloxacin_MG1063_t120 | 1 | -45.15 |
| 304 | K12_t90 | 3 | -45.89 |
| 305 | har_S4_R_noIPTG | 3 | -51.28 |
|  |  |  |  |
