# Supplementary material for: Query Large Scale Microarray Compendium Datasets Using a Model-Based Bayesian Approach with Variable Selection
Source: PLoS One. 2009 Feb 13;4(2):e4495. doi: 10.1371/journal.pone.0004495 (PMC2637418; doi:10.1371/journal.pone.0004495)
Supplement: Table S4 — (0.02 MB DOC) [file pone.0004495.s006.doc]

**Table S4.** Information on the 57 potential Lrp target genes identified by BEST in the 300-gene test set extracted from the *E. coli* compendium.

|  |  |  |  |  |  |
| --- | --- | --- | --- | --- | --- |
| Rank | Gene Name ^a^ | Log Bayes Ratio | positive/negative ^b^ | RegulonDB ^c^ | CLR ^d^ |
| 1 | serA | 187.8 |  | X | X |
| 2 | gltD | 182.97 |  | X | X |
| 3 | metE | 180.95 |  |  | X |
| 4 | leuL | 178.03 |  | X | X |
| 5 | leuD | 175.01 |  | X | X |
| 6 | leuA | 174.43 |  | X | X |
| 7 | gltB | 173.3 |  | X |  |
| 8 | livG | 172.8 |  | X | X |
| 9 | livJ | 172.44 |  | X |  |
| 10 | ilvE | 172.35 |  | X |  |
| 11 | ompT ^†^ | 169.81 |  |  | X |
| 12 | pyrI | 169.77 |  |  |  |
| 13 | livK | 169.16 |  | X | X |
| 14 | ilvH | 168.41 |  | X | X |
| 15 | leuC | 168.1 |  | X | X |
| 16 | ilvI | 168 |  | X |  |
| 17 | gcvB | 166.47 | negative |  |  |
| 18 | serC | 164.09 |  | X | X |
| 19 | livM | 163.56 |  | X |  |
| 20 | leuB | 163.18 |  | X | X |
| 21 | pyrB | 161.91 |  |  |  |
| 22 | yagU ^†^ | 158.71 |  |  | X |
| 23 | aroA | 158.46 |  | X | X |
| 24 | cysD | 158.39 |  |  |  |
| 25 | ilvD | 157.84 |  | X |  |
| 26 | lysU | 157.56 | negative | X |  |
| 27 | livH | 157.28 |  | X | X |
| 28 | livF | 155.74 |  | X | X |
| 29 | stpA | 153.87 |  | X |  |
| 30 | cysK | 152.23 |  |  |  |
| 31 | pheL | 151.32 |  |  |  |
| 32 | tnaC | 149.8 | negative |  |  |
| 33 | dppA | 148.7 |  |  |  |
| 34 | cysN | 147.61 |  |  |  |
| 35 | kbl | 145.76 | negative | X |  |
| 36 | treC | 143.28 | negative |  |  |
| 37 | ilvL | 142.44 |  | X |  |
| 38 | tdh | 140.63 | negative | X |  |
| 39 | pyrL | 140.4 |  |  |  |
| 40 | ilvC | 139.63 |  |  |  |
| 41 | sdaA | 138.77 | negative | X |  |
| 42 | sdaC | 136.96 | negative |  |  |
| 43 | ilvA | 136.74 |  | X |  |
| 44 | thrL | 135.9 |  |  |  |
| 45 | hisL | 135.55 |  |  |  |
| 46 | yeeD | 133.82 |  |  |  |
| 47 | ilvM | 131.85 |  | X | X |
| 48 | treB | 130.2 | negative |  |  |
| 49 | ompF | 129.21 |  | X |  |
| 50 | fdoG | 127.87 | negative |  |  |
| 51 | oppA | 127.26 |  | X |  |
| 52 | oppB | 124.59 |  | X |  |
| 53 | rmf | 122.65 |  |  |  |
| 54 | oppF | 122.24 |  | X |  |
| 55 | ynaJ | 118.39 |  |  |  |
| 56 | ilvG | 113.06 |  | X |  |
| 57 | sroF | 109.54 |  |  |  |
|  |  |  |  |  |  |

^a^ Genes displayed here are sorted by the Log Bayes ratio (target gene versus non-target gene).

^b^ Blank mean the target gene shows the same pattern as the query gene. Negative means the target gene shows the inversed pattern as the query gene.

^c^ BEST indentifies 33 genes among 61 target genes in RegulonDB. “X” indicates that the predicted gene is in the RegulonDB target set.

^d^ “X” indicates that the gene is predicted by CLR as a target gene.

^†^ Previously unknown targets of Lrp, experimentally verified by ChIP (Faith et al. 2007).
