# Supplementary material for: Query Large Scale Microarray Compendium Datasets Using a Model-Based Bayesian Approach with Variable Selection
Source: PLoS One. 2009 Feb 13;4(2):e4495. doi: 10.1371/journal.pone.0004495 (PMC2637418; doi:10.1371/journal.pone.0004495)
Supplement: Table S5 — (0.02 MB DOC) [file pone.0004495.s007.doc]

**Table S5.** Information on the 27 potential PdhR target genes identified by BEST in the 100-gene test set extracted from the *E. coli* compendium.

|  |  |  |  |  |  |
| --- | --- | --- | --- | --- | --- |
| Rank | Gene Name ^a^ | Log Bayes Ratio | positive/negative ^b^ | RegulonDB ^c^ | CLR ^d^ |
| 1 | recN | 348.48 |  |  |  |
| 2 | intE | 342.08 |  |  |  |
| 3 | recA | 337.20 |  |  |  |
| 4 | tisB | 336.43 |  |  |  |
| 5 | xisE | 332.99 |  |  |  |
| 6 | araB | 330.06 |  |  |  |
| 7 | araA | 328.56 |  |  |  |
| 8 | sulA | 328.12 |  |  |  |
| 9 | araD | 327.70 |  |  |  |
| 10 | ymfJ | 322.03 |  |  |  |
| 11 | ymfT | 317.29 |  |  |  |
| 12 | ymfL | 316.94 |  |  |  |
| 13 | araE | 314.20 |  |  |  |
| 14 | murC | 307.50 |  |  | X |
| 15 | ftsW | 306.20 |  |  | X |
| 16 | murD | 304.53 |  |  | X |
| 17 | ndh | 298.38 |  |  | X |
| 18 | aceE | 283.28 |  | X |  |
| 19 | aceF | 279.67 |  | X |  |
| 20 | uspE | 275.72 | negative |  |  |
| 21 | proV | 274.19 |  |  |  |
| 22 | cspD | 267.67 | negative |  |  |
| 23 | isrB | 263.68 |  |  |  |
| 24 | spf | 248.58 |  |  |  |
| 25 | cspA | 239.93 |  |  |  |
| 26 | tisA | 221.34 |  |  |  |
| 27 | aceA | 76.68 | negative |  |  |
|  |  |  |  |  |  |

^a^ Genes displayed here are sorted by the Log Bayes ratio (target gene versus non-target gene).

^b^ Blank indicates that the target gene shows the same pattern as the query gene. Negative indicates that the target gene shows the inversed pattern as the query gene.

^c^ BEST indentifies two genes among five target genes in RegulonDB. “X” indicates that the predicted gene is in the RegulonDB target set.

^d^ “X” indicates that the gene is predicted by CLR as a target gene.
