# Supplementary material for: Query Large Scale Microarray Compendium Datasets Using a Model-Based Bayesian Approach with Variable Selection
Source: PLoS One. 2009 Feb 13;4(2):e4495. doi: 10.1371/journal.pone.0004495 (PMC2637418; doi:10.1371/journal.pone.0004495)
Supplement: Table S6 — (0.02 MB DOC) [file pone.0004495.s008.doc]

**Table S6.** Information on the 31 potential FecI target genes identified by BEST in the 100-gene test set extracted from the *E. coli* compendium.

|  |  |  |  |  |  |
| --- | --- | --- | --- | --- | --- |
| Rank | Gene Name ^a^ | Log Bayes Ratio | positive/negative ^b^ | RegulonDB ^c^ | CLR ^d^ |
| 1 | ymfT | 206.62 |  |  |  |
| 2 | ymfJ | 200.23 |  |  |  |
| 3 | ymfL | 196.67 |  |  |  |
| 4 | araD | 189.08 |  |  |  |
| 5 | xisE | 185.50 |  |  |  |
| 6 | araB | 185.40 |  |  |  |
| 7 | araA | 182.57 |  |  |  |
| 8 | recN | 177.90 |  |  |  |
| 9 | araE | 177.45 |  |  |  |
| 10 | tisB | 146.18 |  |  |  |
| 11 | tisA | 144.54 |  |  |  |
| 12 | sulA | 133.96 |  |  |  |
| 13 | recA | 118.89 |  |  |  |
| 14 | proV | 81.52 |  |  |  |
| 15 | fecE | 72.40 |  | X |  |
| 16 | fecB | 67.88 |  | X |  |
| 17 | isrB | 63.99 |  |  |  |
| 18 | fecD | 63.63 |  | X |  |
| 19 | fecC | 63.13 |  | X |  |
| 20 | fecA | 62.00 |  | X |  |
| 21 | fhuF | 54.12 |  |  | X |
| 22 | ybaN | 53.60 |  |  | X |
| 23 | exbB | 46.24 |  |  | X |
| 24 | fhuA | 44.64 |  |  | X |
| 25 | exbD | 43.74 |  |  | X |
| 26 | fecR | 43.29 |  | X | X |
| 27 | bfd | 33.30 |  |  |  |
| 28 | micF | 32.89 |  |  |  |
| 29 | spf | 29.53 |  |  |  |
| 30 | cspA | 19.14 |  |  |  |
| 31 | entB | -0.34 |  |  | X |
|  |  |  |  |  |  |
|  |  |  |  |  |  |

^a^ Genes displayed here are sorted by the Log Bayes ratio (target gene versus non-target gene).

^b^ Blank indicates that the target gene shows the same pattern as the query gene. Negative indicates that the target gene shows the inversed pattern as the query gene.

^c^ BEST indentifies all six genes among six target genes in RegulonDB. “X” indicates that the predicted gene is in the RegulonDB target set.

^d^ “X” indicates that the gene is predicted by CLR as a target gene.
