# Supplementary material for: Query Large Scale Microarray Compendium Datasets Using a Model-Based Bayesian Approach with Variable Selection
Source: PLoS One. 2009 Feb 13;4(2):e4495. doi: 10.1371/journal.pone.0004495 (PMC2637418; doi:10.1371/journal.pone.0004495)
Supplement: Table S7 — (0.02 MB DOC) [file pone.0004495.s009.doc]

**Table S7.** Information on the 31 potential LexA target genes identified by BEST in the 100-gene test set extracted from the *E. coli* compendium

|  |  |  | |  | |  | |  | |  |
| --- | --- | --- | --- | --- | --- | --- | --- | --- | --- | --- |
| Rank | Gene Name ^a^ | Log Bayes Ratio | | positive/negative ^b^ | | RegulonDB ^c^ | | CLR ^d^ | |  |
| 1 | dinF | 370.20 | |  | | X | | X | |  |
| 2 | araB | 365.72 | |  | |  | |  | |  |
| 3 | araA | 365.42 | |  | |  | |  | |  |
| 4 | araE | 359.80 | |  | |  | |  | |  |
| 5 | araD | 358.89 | |  | |  | |  | |  |
| 6 | ymfJ | 349.63 | |  | |  | |  | |  |
| 7 | ymfL | 347.87 | |  | |  | |  | |  |
| 8 | recN | 347.63 | |  | | X | | X | |  |
| 9 | xisE | 339.91 | |  | |  | |  | |  |
| 10 | yebG | 333.06 | |  | |  | | X | |  |
| 11 | ymfT | 332.53 | |  | |  | |  | |  |
| 12 | dinI | 320.63 | |  | |  | | X | |  |
| 13 | recX | 318.60 | |  | |  | |  | |  |
| 14 | umuD | 316.82 | |  | | X | | X | |  |
| 15 | tisB | 314.57 | |  | |  | |  | |  |
| 16 | yafN | 314.26 | |  | |  | | X | |  |
| 17 | tisA | 312.63 | |  | |  | |  | |  |
| 18 | dinD | 312.63 | |  | |  | | X | |  |
| 19 | uvrA | 310.18 | |  | | X | | X | |  |
| 20 | dinG | 308.19 | |  | |  | | X | |  |
| 21 | yafO | 306.66 | |  | |  | | X | |  |
| 22 | sulA | 305.85 | |  | | X | | X | |  |
| 23 | polB | 299.75 | |  | | X | |  | |  |
| 24 | recA | 292.55 | |  | | X | | X | |  |
| 25 | umuC | 289.20 | |  | | X | | X | |  |
| 26 | dinB | 282.91 | |  | |  | |  | |  |
| 27 | bssS | 262.57 | |  | |  | |  | |  |
| 28 | ssb | 262.39 | |  | | X | |  | |  |
| 29 | uvrD | 259.91 | |  | | X | |  | |  |
| 30 | yebF | 242.58 | |  | |  | | X | |  |
| 31 | uspE | 239.61 | | negative | |  | |  | |  |
|  |  |  | |  | |  | |  | |  |
|  |  | |  | |  | |  | |  | |

^a^ Genes displayed here are sorted by the Log Bayes ratio (target gene versus non-target gene).

^b^ Blank indicates that the target gene shows the same pattern as the query gene. Negative indicates that the target gene shows the inversed pattern as the query gene.

^c^ BEST indentifies ten genes among 16 target genes in RegulonDB. “X” indicates that the predicted gene is in the RegulonDB target set.

^d^ “X” indicates that the gene is predicted by CLR as a target gene.
