# Supplementary material for: Query Large Scale Microarray Compendium Datasets Using a Model-Based Bayesian Approach with Variable Selection
Source: PLoS One. 2009 Feb 13;4(2):e4495. doi: 10.1371/journal.pone.0004495 (PMC2637418; doi:10.1371/journal.pone.0004495)
Supplement: Table S8 — (0.02 MB DOC) [file pone.0004495.s010.doc]

**Table S8.** Information on the 54 potential FlhC target genes identified by BEST in the 200-gene test set extracted from the *E. coli* compendium

|  |  |  |  |  |  |
| --- | --- | --- | --- | --- | --- |
| Rank | Gene Name ^a^ | Log Bayes Ratio | positive/negative ^b^ | RegulonDB ^c^ | CLR ^d^ |
| 1 | flgE | 422.41 |  | X | X |
| 2 | fliA | 417.80 |  | X | X |
| 3 | flgC | 415.00 |  | X | X |
| 4 | flgB | 412.97 |  | X | X |
| 5 | flgG | 406.95 |  | X | X |
| 6 | flgH | 406.29 |  | X | X |
| 7 | flgD | 403.29 |  | X | X |
| 8 | flhD | 401.18 |  |  | X |
| 9 | motB | 398.98 |  |  | X |
| 10 | fliL | 398.54 |  | X | X |
| 11 | fliN | 398.03 |  | X | X |
| 12 | flgI | 397.19 |  | X | X |
| 13 | flgK | 396.32 |  |  | X |
| 14 | flgA | 396.03 |  | X | X |
| 15 | fliK | 389.85 |  | X | X |
| 16 | fliM | 388.79 |  | X | X |
| 17 | flgF | 388.45 |  | X | X |
| 18 | motA | 387.71 |  |  | X |
| 19 | cheA | 386.19 |  |  | X |
| 20 | cheW | 385.20 |  |  | X |
| 21 | fliZ | 384.50 |  | X | X |
| 22 | fliJ | 384.25 |  | X | X |
| 23 | flgM | 382.36 |  |  | X |
| 24 | fliF | 382.00 |  | X | X |
| 25 | flgN | 380.73 |  |  | X |
| 26 | fliS | 380.29 |  |  | X |
| 27 | cheY | 378.15 |  |  | X |
| 28 | flgJ | 372.04 |  | X | X |
| 29 | cheZ | 371.92 |  |  | X |
| 30 | cheR | 371.27 |  |  | X |
| 31 | yecR | 370.70 |  |  | X |
| 32 | cheB | 369.11 |  |  | X |
| 33 | fliG | 367.55 |  | X | X |
| 34 | fliC | 366.59 |  |  | X |
| 35 | flgL | 366.38 |  |  | X |
| 36 | fliH | 362.55 |  | X | X |
| 37 | fliD | 360.82 |  |  | X |
| 38 | fliP | 356.28 |  | X | X |
| 39 | fliQ | 350.16 |  | X | X |
| 40 | tar | 347.51 |  |  | X |
| 41 | fliI | 345.24 |  | X | X |
| 42 | ycgR | 344.20 |  |  | X |
| 43 | tap | 338.41 |  |  | X |
| 44 | fliE | 326.37 |  | X | X |
| 45 | flxA | 325.67 |  |  | X |
| 46 | fliO | 324.78 |  | X | X |
| 47 | ymdA | 314.15 |  |  | X |
| 48 | flhA | 301.22 |  | X | X |
| 49 | flhE | 300.16 |  | X | X |
| 50 | flhB | 290.68 |  | X | X |
| 51 | fliR | 275.89 |  | X | X |
| 52 | yhjH | 272.24 |  |  | X |
| 53 | tsr | 255.05 |  |  | X |
| 54 | yjdA | 206.14 |  |  |  |
|  |  |  |  |  |  |

^a^ Genes displayed here are sorted by the Log Bayes ratio (target gene versus non-target gene).

^b^ Blank indicates that the target gene shows the same pattern as the query gene. Negative indicates that the target gene shows the inversed pattern as the query gene.

^c^ BEST indentifies 29 genes among 30 target genes in RegulonDB. “X” indicates that the predicted gene is in the RegulonDB target set.

^d^ “X” indicates that the gene is predicted by CLR as a target gene.
