# Supplementary material for: Query Large Scale Microarray Compendium Datasets Using a Model-Based Bayesian Approach with Variable Selection
Source: PLoS One. 2009 Feb 13;4(2):e4495. doi: 10.1371/journal.pone.0004495 (PMC2637418; doi:10.1371/journal.pone.0004495)
Supplement: Table S9 — (0.02 MB DOC) [file pone.0004495.s011.doc]

**Table S9.** Information on the 67 potential FlhD target genes identified by BEST in the 200-gene test set extracted from the *E. coli* compendium

|  |  |  |  |  |  |
| --- | --- | --- | --- | --- | --- |
| Rank | Gene Name ^a^ | Log Bayes Ratio | positive/negative ^b^ | RegulonDB ^c^ | CLR ^d^ |
| 1 | flhC | 333.41 |  |  | X |
| 2 | flgE | 312.18 |  | X | X |
| 3 | flgB | 311.50 |  | X | X |
| 4 | flgH | 311.46 |  | X | X |
| 5 | fliA | 311.18 |  | X | X |
| 6 | flgC | 310.34 |  | X | X |
| 7 | flgG | 307.33 |  | X | X |
| 8 | flgD | 299.71 |  | X | X |
| 9 | flgK | 299.55 |  |  | X |
| 10 | flgI | 299.12 |  | X | X |
| 11 | fliZ | 298.07 |  | X | X |
| 12 | flgA | 296.58 |  | X | X |
| 13 | motB | 294.72 |  |  | X |
| 14 | cheW | 293.40 |  |  | X |
| 15 | flgF | 293.22 |  | X | X |
| 16 | fliN | 292.68 |  | X | X |
| 17 | fliL | 292.29 |  | X | X |
| 18 | flgN | 291.93 |  |  | X |
| 19 | fliF | 290.99 |  | X | X |
| 20 | flgM | 290.12 |  |  | X |
| 21 | fliM | 288.74 |  | X | X |
| 22 | cheA | 288.54 |  |  | X |
| 23 | fliK | 285.84 |  | X | X |
| 24 | fliS | 282.94 |  |  | X |
| 25 | motA | 282.93 |  |  | X |
| 26 | yecR | 282.39 |  |  | X |
| 27 | flgL | 281.34 |  |  | X |
| 28 | fliJ | 279.79 |  | X | X |
| 29 | fliC | 275.71 |  |  | X |
| 30 | flgJ | 275.32 |  | X | X |
| 31 | fliD | 271.73 |  |  | X |
| 32 | cheR | 271.62 |  |  | X |
| 33 | fliP | 270.94 |  | X | X |
| 34 | fliG | 270.80 |  | X | X |
| 35 | cheB | 269.55 |  |  | X |
| 36 | cheY | 269.30 |  |  |  |
| 37 | fliH | 264.98 |  | X |  |
| 38 | tar | 264.64 |  |  | X |
| 39 | cheZ | 264.38 |  |  |  |
| 40 | fliE | 260.07 |  | X | X |
| 41 | fliI | 256.40 |  | X | X |
| 42 | ycgR | 251.77 |  |  | X |
| 43 | flxA | 251.29 |  |  |  |
| 44 | fliQ | 251.26 |  | X | X |
| 45 | flhE | 231.94 |  | X |  |
| 46 | fliO | 229.43 |  | X | X |
| 47 | flhB | 222.84 |  | X |  |
| 48 | flhA | 221.21 |  | X | X |
| 49 | ymdA | 218.56 |  |  | X |
| 50 | fliR | 196.03 |  | X | X |
| 51 | tsr | 192.51 |  |  | X |
| 52 | yibT | 191.41 |  |  |  |
| 53 | yhjH | 191.29 |  |  | X |
| 54 | yjbJ | 188.62 |  |  |  |
| 55 | hdeB | 187.86 |  |  |  |
| 56 | slp | 184.57 |  |  |  |
| 57 | ompF | 181.05 |  |  |  |
| 58 | micF | 178.49 | negative |  |  |
| 59 | gadE | 178.32 | negative |  |  |
| 60 | hdeA | 177.08 |  |  |  |
| 61 | hdeD | 176.26 |  |  |  |
| 62 | gadX | 173.90 |  |  |  |
| 63 | gadB | 171.77 |  |  |  |
| 64 | gadA | 165.48 |  |  |  |
| 65 | yjdA | 148.57 |  |  |  |
| 66 | bssS | 122.36 |  |  |  |
| 67 | ygiW | 109.00 |  |  |  |
|  |  |  |  |  |  |

^a^ Genes displayed here are sorted by the Log Bayes ratio (target gene versus non-target gene).

^b^ Blank indicates that the target gene shows the same pattern as the query gene. Negative indicates that the target gene shows the inversed pattern as the query gene.

^c^ BEST indentifies 29 genes among 46 target genes in RegulonDB. “X” indicates that the predicted gene is in the RegulonDB target set.

^d^ “X” indicates that the gene is predicted by CLR as a target gene.
