# Supplementary material for: Query Large Scale Microarray Compendium Datasets Using a Model-Based Bayesian Approach with Variable Selection
Source: PLoS One. 2009 Feb 13;4(2):e4495. doi: 10.1371/journal.pone.0004495 (PMC2637418; doi:10.1371/journal.pone.0004495)
Supplement: Table S10 — (0.02 MB DOC) [file pone.0004495.s012.doc]

**Table S10.** Information on the 56 potential FliA target genes identified by BEST in the 200-gene test set extracted from the *E. coli* compendium

|  |  |  |  |  |  |
| --- | --- | --- | --- | --- | --- |
| Rank | Gene Name ^a^ | Log Bayes Ratio | positive/negative ^b^ | RegulonDB ^c^ | CLR ^d^ |
| 1 | fliZ | 524.28 |  | X | X |
| 2 | flgE | 520.41 |  | X | X |
| 3 | flgC | 518.79 |  | X | X |
| 4 | flgB | 510.76 |  | X | X |
| 5 | flgG | 506.72 |  | X | X |
| 6 | flgD | 506.65 |  | X | X |
| 7 | flgN | 503.87 |  |  | X |
| 8 | flgK | 503.51 |  |  | X |
| 9 | flgH | 490.47 |  | X | X |
| 10 | flgM | 488.91 |  |  | X |
| 11 | fliD | 484.75 |  | X | X |
| 12 | cheW | 484.68 |  | X | X |
| 13 | cheA | 480.61 |  | X | X |
| 14 | motB | 470.69 |  | X | X |
| 15 | motA | 469.88 |  | X | X |
| 16 | flgL | 469.11 |  | X | X |
| 17 | flgA | 467.86 |  |  | X |
| 18 | fliK | 463.84 |  | X | X |
| 19 | fliS | 459.14 |  | X | X |
| 20 | fliN | 456.92 |  | X | X |
| 21 | flgF | 456.74 |  | X | X |
| 22 | cheZ | 453.19 |  | X | X |
| 23 | cheR | 452.47 |  | X | X |
| 24 | fliL | 452.27 |  | X | X |
| 25 | fliJ | 452.03 |  | X | X |
| 26 | flgI | 448.03 |  | X | X |
| 27 | cheB | 443.04 |  | X | X |
| 28 | tar | 439.80 |  |  | X |
| 29 | fliC | 439.60 |  |  | X |
| 30 | fliF | 435.47 |  | X | X |
| 31 | fliM | 431.91 |  | X | X |
| 32 | fliG | 429.41 |  | X | X |
| 33 | cheY | 425.70 |  | X | X |
| 34 | flgJ | 423.63 |  | X | X |
| 35 | fliP | 420.70 |  | X | X |
| 36 | yecR | 418.55 |  |  | X |
| 37 | ycgR | 415.26 |  |  | X |
| 38 | tap | 414.20 |  | X | X |
| 39 | fliQ | 404.20 |  | X | X |
| 40 | fliH | 389.79 |  | X | X |
| 41 | flxA | 377.76 |  | X | X |
| 42 | fliT | 369.77 |  | X | X |
| 43 | ymdA | 364.62 |  |  | X |
| 44 | fliO | 361.51 |  | X | X |
| 45 | fliI | 355.28 |  | X | X |
| 46 | fliE | 354.40 |  | X | X |
| 47 | flhC | 348.31 |  |  | X |
| 48 | flhB | 335.53 |  | X | X |
| 49 | flhE | 321.02 |  | X | X |
| 50 | flhA | 317.85 |  | X | X |
| 51 | fliR | 312.35 |  | X | X |
| 52 | yhjH | 310.92 |  |  | X |
| 53 | flhD | 305.93 |  |  | X |
| 54 | tsr | 281.05 |  |  | X |
| 55 | ves | 233.09 |  |  | X |
| 56 | yjdA | 187.37 |  |  | X |
|  |  |  |  |  |  |

^a^ Genes displayed here are sorted by the Log Bayes ratio (target gene versus non-target gene).

^b^ Blank indicates that the target gene shows the same pattern as the query gene. Negative indicates that the target gene shows the inversed pattern as the query gene.

^c^ BEST indentifies 41 among 42 target genes in RegulonDB. “X” indicates that the predicted gene is in the RegulonDB target set.

^d^ “X” indicates that the gene is predicted by CLR as a target gene.
