# Supplementary figures and images for: Query Large Scale Microarray Compendium Datasets Using a Model-Based Bayesian Approach with Variable Selection
Source: PLoS One. 2009 Feb 13;4(2):e4495. doi: 10.1371/journal.pone.0004495 (PMC2637418; doi:10.1371/journal.pone.0004495)

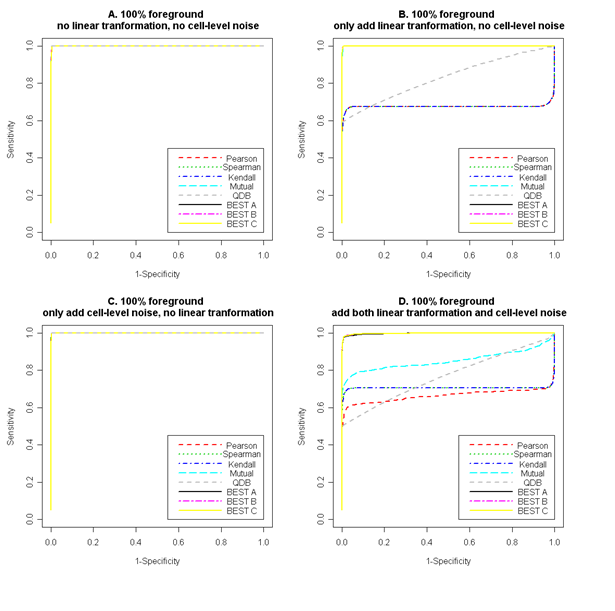

Supplement: Figure S1 — ROC curves for various query methods when applying to synthetic datasets simulated under different settings and when there are 100% foreground columns. BEST A default setting; BEST B allowing exclusion of individual cells from the foreground; BEST C fixing the indicator variables of five true target genes and five true experimental conditions as 1. A. No linear transformation nor cell-level noise. B. With linear transformation only. C. With cell-level noise only. D. With both linear transformation and cell-level noise. (0.08 MB TIF) [file pone.0004495.s013.tif]

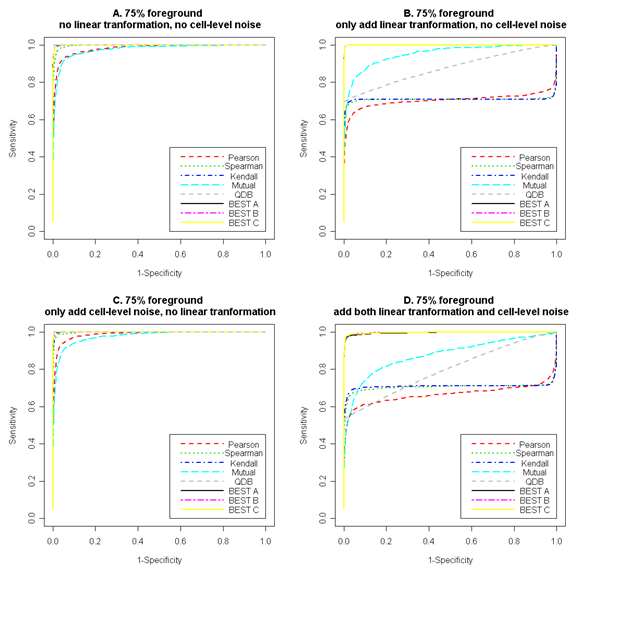

Supplement: Figure S2 — ROC curves for various query methods when applying to synthetic datasets simulated under different settings and when there are 75% foreground columns. BEST A default setting; BEST B allowing exclusion of individual cells from the foreground; BEST C fixing the indicator variables of five true target genes and five true experimental conditions as 1. A. No linear transformation nor cell-level noise. B. With linear transformation only. C. With cell-level noise only. D. With both linear transformation and cell-level noise. (0.09 MB TIF) [file pone.0004495.s014.tif]

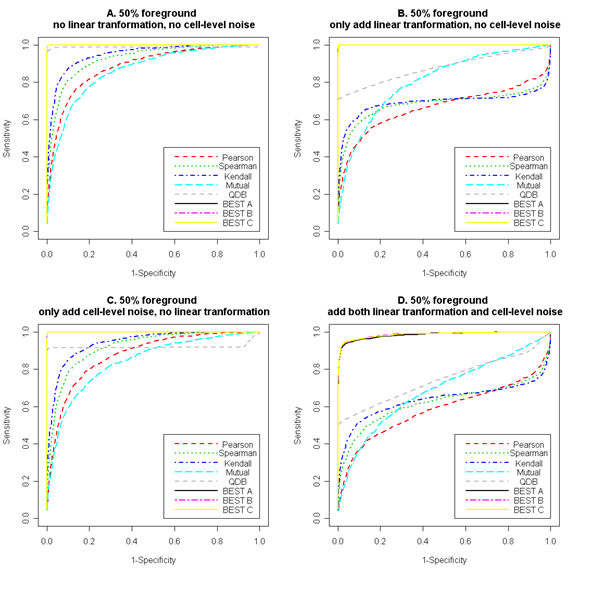

Supplement: Figure S3 — ROC curves for various query methods when applying to synthetic datasets simulated under different settings and when there are 50% foreground columns. BEST A default setting; BEST B allowing exclusion of individual cells from the foreground; BEST C fixing the indicator variables of five true target genes and five true experimental conditions as 1. A. No linear transformation nor cell-level noise. B. With linear transformation only. C. With cell-level noise only. D. With both linear transformation and cell-level noise. (0.08 MB TIF) [file pone.0004495.s015.tif]

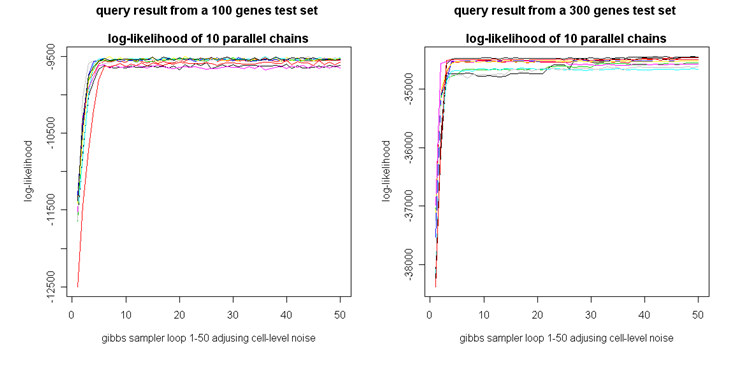

Supplement: Figure S4 — Log-likelihood trace plots of the ten parallel chains resulted from the BEST run on 100-gene and 300-gene test sets selected from the E. coli microarray compendium. (0.06 MB TIF) [file pone.0004495.s016.tif]

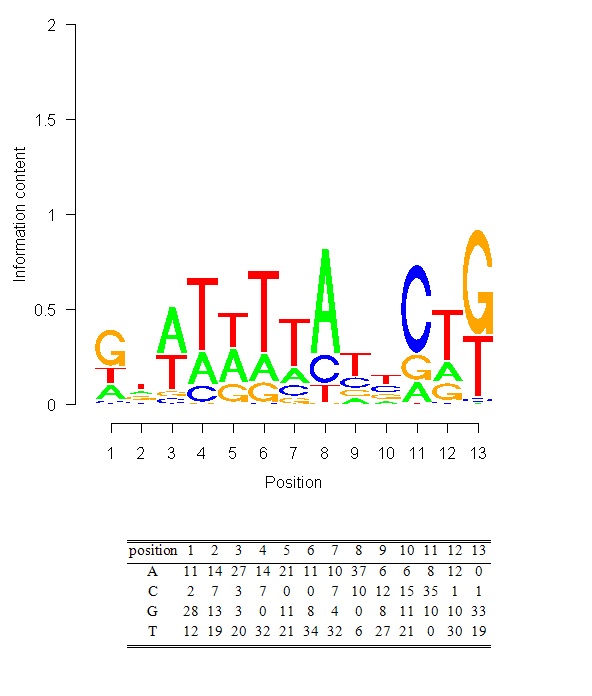

Supplement: Figure S5 — Sequence logo plot [1] and position specific weight matrix (PSWM) for the motif of transcription factor Lrp. Lrp motif is downloaded from regulonDB: http://regulondb.ccg.unam.mx/data/Matrix_AlignmentSet.txt. The logo plot was generated by the seqLogo program [4]. (0.03 MB TIF) [file pone.0004495.s017.tif]

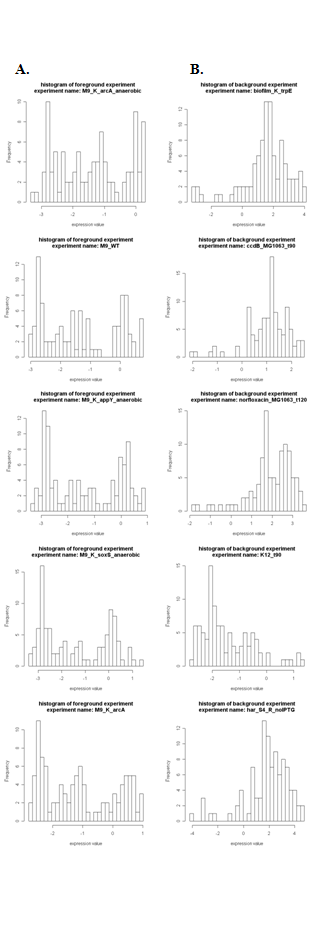

Supplement: Figure S6 — Boxplots of Pearson correlation coefficients. A. Boxplots of Pearson correlations between expression profiles of the 61 experimentally verified Lrp target genes and Lrp. The left one summarize correlations measured in the 162 background experiments and the right one summarize correlations measured in the 143 foreground experiments. A paired t-test comparing the two sets of correlation coefficients returns a p-value of 0.0079. B. Boxplots of Pearson correlations between expression profiles of the 28 genes BEST indentified as Lrp target. The left one summarize correlations measured in the 162 background experiments and the right one summarize correlations measured in the 143 foreground experiments. A paired t-test comparing the two sets of correlation coefficients returns a p-value of 1.948×10^–12^. (0.08 MB TIF) [file pone.0004495.s018.tif]

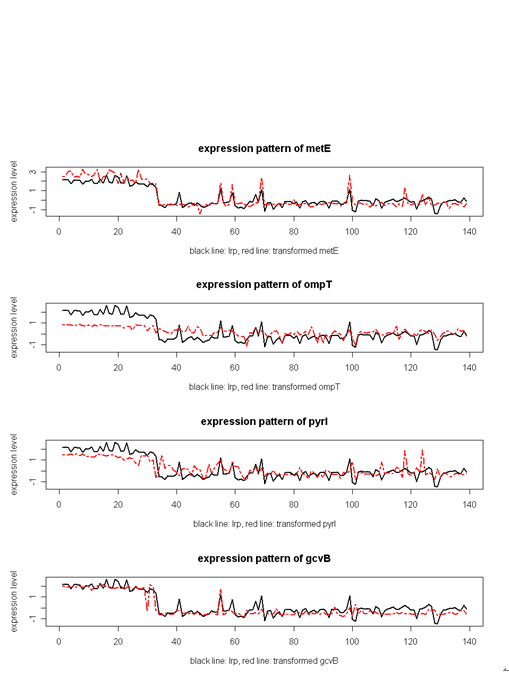

Supplement: Figure S8 — Trace plots of 24 predicted Lrp target genes identified by BEST that are not in the RegulonDB target set (Part 1). Black lines indicate the query gene-Lrp, the red line indicate the potential target genes. Only the 139 foreground experimental conditions identified by BEST were shown in these plots (0.07 MB TIF) [file pone.0004495.s020.tif]

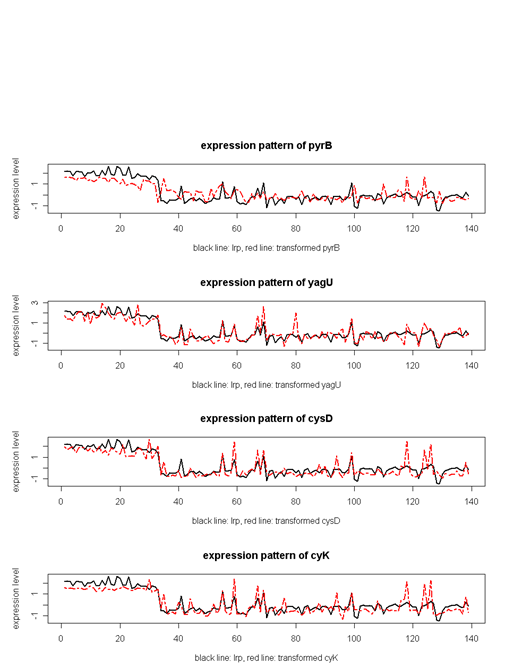

Supplement: Figure S9 — Trace plots of 24 predicted Lrp target genes identified by BEST that are not in the RegulonDB target set (Part 2). Black lines indicate the query gene-Lrp, the red line indicate the potential target genes. Only the 139 foreground experimental conditions identified by BEST were shown in these plots (0.08 MB TIF) [file pone.0004495.s021.tif]

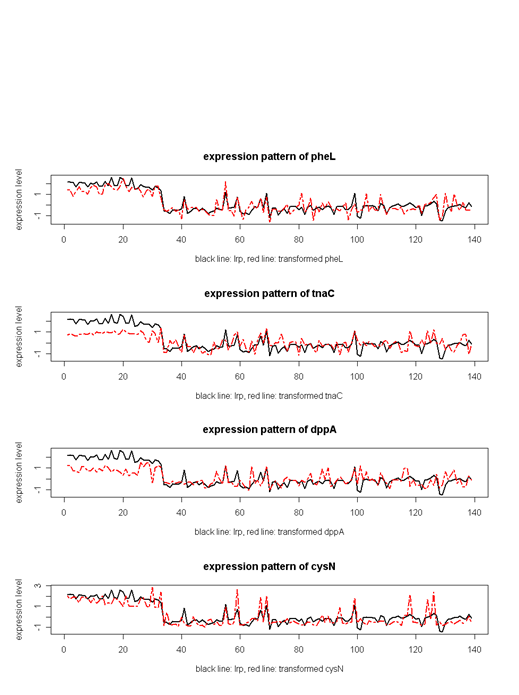

Supplement: Figure S10 — Trace plots of 24 predicted Lrp target genes identified by BEST that are not in the RegulonDB target set (Part 3). Black lines indicate the query gene-Lrp, the red line indicate the potential target genes. Only the 139 foreground experimental conditions identified by BEST were shown in these plots (0.08 MB TIF) [file pone.0004495.s022.tif]

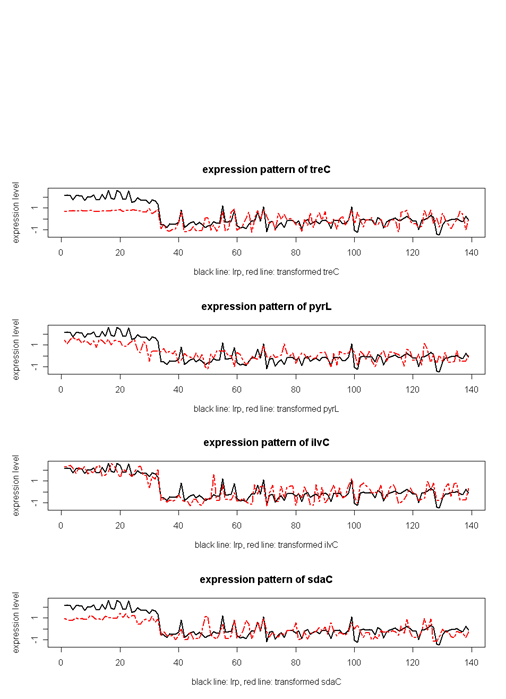

Supplement: Figure S11 — Trace plots of 24 predicted Lrp target genes identified by BEST that are not in the RegulonDB target set (Part 4). Black lines indicate the query gene-Lrp, the red line indicate the potential target genes. Only the 139 foreground experimental conditions identified by BEST were shown in these plots (0.08 MB TIF) [file pone.0004495.s023.tif]

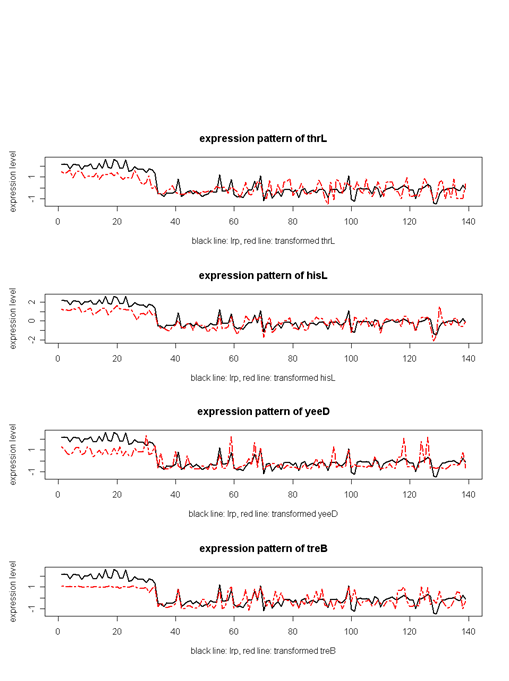

Supplement: Figure S12 — Trace plots of 24 predicted Lrp target genes identified by BEST that are not in the RegulonDB target set (Part 5). Black lines indicate the query gene-Lrp, the red line indicate the potential target genes. Only the 139 foreground experimental conditions identified by BEST were shown in these plots (0.08 MB TIF) [file pone.0004495.s024.tif]

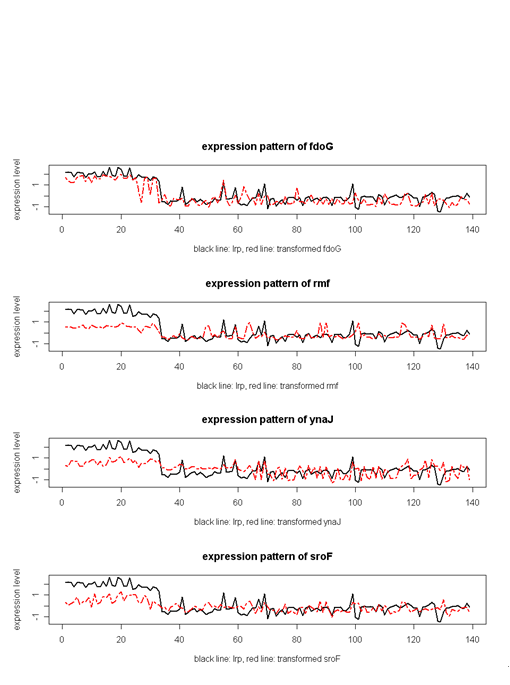

Supplement: Figure S13 — Trace plots of 24 predicted Lrp target genes identified by BEST that are not in the RegulonDB target set (Part 6). Black lines indicate the query gene-Lrp, the red line indicate the potential target genes. Only the 139 foreground experimental conditions identified by BEST were shown in these plots (0.07 MB TIF) [file pone.0004495.s025.tif]

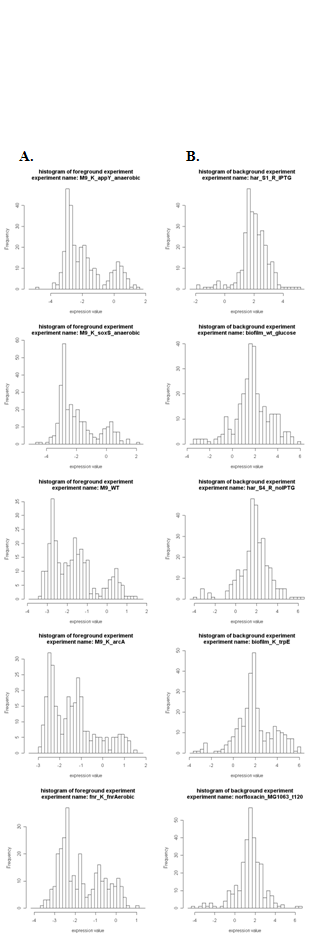

Supplement: Figure S14 — A. Histograms of expression profile differences (zij) in the top five experimental conditions (foreground). B. Histogram of expression profile differences (zij) in the bottom five experimental conditions (background). Data used here is the 300-gene test set selected from the E. coli microarray compendium (0.08 MB TIF) [file pone.0004495.s026.tif]
